# On the Relations between Atmospheric Pollution in Urban and Rural Localities and Mortality from Cancer, Bronchitis and Pneumonia, with Particular Reference to 3:4 Benzopyrene, Beryllium, Molybdenum, Vanadium and Arsenic

**DOI:** 10.1038/bjc.1960.45

**Published:** 1960-09

**Authors:** Percy Stocks


VOL. XIV SEPTEMBER, 1960 NO. 3

### ON THE RELATIONS BETWEEN ATMOSPHERIC POLLUTION IN URBAN AND RURAL LOCALITIES AND MORTALITY FROM CANCER, BRONCHITIS AND PNEUMONIA, WITH PARTICULAR REFERENCE TO 3 : 4 BENZOPYRENE, BERYLLIUM, MOLYBDENUM, VANADIUM AND ARSENIC

PERCY STOCKS

*From Colwyn Bay, North Wales*

Received for publication July 12, 1960

*History and purpose of study*

IN 1954 arrangements were made jointly with the British Empire Cancer Campaign, Medical Research Council and Department of Scientific and Industrial Research to commence an investigation into the amounts of smoke, certain polycyclic hydrocarbons and trace elements in the atmosphere at a number of places in North Wales and Liverpool Hospital Region with a view to relating them with the mortality from Cancer in the areas concerned. In the first instance 10 instruments for continuous collection of smoke on filter papers were placed in urban and rural situations. These were equipped with meters to measure the volume of air passing through, and the filter papers were changed at weekly or monthly intervals and sent for hydrocarbon analysis to the M.R.C. Group at St. Bartholomew's Hospital, London, and for trace element analysis to the Fuel Research Station of the D.S.I.R. at East Greenwich. Results of the measurements of 3 : 4 benzopyrene, pyrene, 1 : 12 benzoperylene and fluoranthene during 6 months from October, 1954, were published in 1955 (Stocks and Campbell, 1955) and results for two years together with data concerning trace elements during 1955 were given in 1958 (Stocks, 1958*a*).

In the autumn of 1956 filters were installed at 13 more places in North Wales, Liverpool and Lancashire, including two in the Mersey Tunnel, two in bus and motor garages, and one in an office, each with an outside control. Hydrocarbon analyses for these and the subsequent series were made by Mr. B. T. Commins of the M.R.C. Group at the Dunn Laboratory. The filters were accompanied by high-speed instruments working alongside the low-speed type to obtain quantities of smoke more adequate for the spectrographic study of trace elements. A report on the smoke and hydrocarbon data from the 23 stations from October 1956 to April 1957 was published with tentative correlations with standardised mortality indices for cancer of the lung and intestine in 15 areas (Stocks, 1958*b*) At the end of 1957, aided by a grant from the Medical Research Council, the mortality data were extended to include bronchitis and pneumonia, and the region was widened by installing low-speed and high-speed filters at five more places in Lancashire (including one in a smokeless zone), at six places in the West Riding of Yorkshire and at four sites in a large steelworks, and of low-speed filters at three places in Tyneside conurbation. This made a total of 41 filter locations as specified in Table I.

TABLE I.—*Localisation of* 41 *Instruments to Collect Smoke Samples for Analysis.*

| County or conurbation | Representative locations | Alternative sites in conurbations | Special locations |
|---|---|---|---|
| Anglesey | Llangefni | — | — |
| Caernarvon | Conway Valley | — | — |
| Merioneth* | Blaenau Ffestiniog* | — | — |
| Denbighshire | Ruthin | — | — |
| | Wrexham | — | — |
| Flintshire | Flint | — | 4 in steelworks.† |
| Merseyside conurbation | Birkenhead | Seaside (Hoylake) | 2 in Mersey tunnel.‡ |
| | Bootle | Riverside‡ | Bus depot (Edge Lane).‡ |
| | Liverpool, Princes Road | Roe Street‡ | Motor car garage.‡ |
| | | Edge Lane‡ | Office interior.* |
| Cheshire | Chester | — | — |
| | Tattenhall | — | — |
| S.E. Lancashire conurbation | Salford, Regent Road | Smokeless zone‡ | — |
| Lancashire | St. Helens | — | — |
| | Warrington‡ | — | — |
| | Ormskirk | — | — |
| | Darwen‡ | — | — |
| | Burnley‡ | — | — |
| | Lancaster‡ | — | — |
| West Yorkshire conurbation | Leeds‡ | — | — |
| | Keighley‡ | — | — |
| | Elland‡ | — | — |
| West Riding | York‡ | — | — |
| | Wetherby‡ | — | — |
| | Ripon‡ | — | — |
| Tynside conurbation | Gateshead‡* | — | — |
| | Newcastle-on-Tyne‡ (Wharncliffe St.)* | Shields Road‡* | — |

* No trace element analyses. † No hydrocarbon analyses.
‡ Analyses of 7 hydrocarbons were made here, and of 4 hydrocarbons elsewhere.

The present paper deals with the representative locations, which alone can be related to mortality rates. Hoylake has not been included with these but considered as a special Merseyside site because many of the residents travel to Liverpool or Birkenhead to work or have retired after working there, and their death rates cannot be fairly related to the local atmospheric conditions. The results from the alternative and special sites have been reported upon in a separate paper (Stocks, Commins and Aubrey, 1960). Data of total smoke and 4 polycyclic hydrocarbons are available at each of the 26 places in the first column of Table I, and amounts of trace elements at 23 of them.

A previous study was made also of the amounts of undissolved deposit and of smoke in those county boroughs of England and Wales and administrative areas of Lancashire and the West Riding of Yorkshire where measurements had been recorded by the D.S.I.R., relating these with standardized mortality ratios for cancer and bronchitis (Stocks, 1959). This has now been extended by addition of more towns, using a more adequate social index, and a similar study has been made of Greater London.

The main purposes of this paper are (1) to establish more firmly the association between smoke and mortality from lung cancer and respiratory disease, and (2) to ascertain which of the polycyclic hydrocarbons and which of the trace elements are concerned in this.

*Evaluation of smoke and polycyclic hydrocarbons in air*

The amount of smoke per cubic metre of air is estimated from a direct photometric measure of the blackness of the circular portion of known area of the filter paper through which a measured volume of air has been drawn by a pump operating continuously for a suitable period. At the end of the period, which may be a week in a highly urbanised district or a month in a rural district, the paper is changed and the results from all the filter papers throughout a year are aggregated when an annual average is required. The blackness readings are converted to milligrams of smoke by means of a standard calibration curve. The calibration was originally carried out on London smoke and it has been found to be reasonably satisfactory for smoke in other large towns in Great Britain. Persistent errors in the absolute values of the smoke concentration determined in this way would not affect comparisons between districts, but there may be local variations in the " blackness " per unit weight of smoke. The standard calibration applies to the usual mixture of domestic smoke plus a little industrial and traffic smoke found in the general atmosphere of British towns and it is unlikely to hold good for special locations where industrial or traffic smoke predominate, such as road tunnels. Any variations of this kind would be liable to reduce the correlation coefficients between smoke and mortality.

The polycyclic hydrocarbons are determined quantitatively and a direct measure of their concentration in the air is obtained by dividing by the volume of air sampled in each case. They can also be expressed as a proportion of the total amount of suspended matter (smoke) as in Table III, and this may be of some importance, quite apart from the absolute concentration in air, but such figures are subject to the errors mentioned above which affect the denominators of the proportions. Sources of error which might depress correlations between amounts of hydrocarbons and mortality rates or social factors include analytical errors, instability of some of the substances leading to partial loss by complex processes such as oxidation or evaporation during the interval between collection of a sample and its analysis, and faults in obtaining representative samples by the choice of sites for the filters. Concerning the analytical technique a modification of this was introduced (Commins, 1958) and applied in 1958–59, but all data used in this paper from previous years have been corrected to allow for the effects of this. As to stability, pyrene and fluoranthene are less stable than 3 : 4 benzopyrene and 1 : 12 benzoperylene and if changes occurred more rapidly in samples from some areas than in samples from elsewhere, correlations between amounts of these substances and mortality rates might be depressed rather more than was the case for the other hydrocarbons. Some internal evidence on this point is provided by the inter-correlations between recorded amounts of the 4 hydrocarbons in the air of 26 localities at the head of Table VI. The first two, 3 : 4 benzopyrene and 1 : 12 benzoperylene, are closely related (0·956), but the last two, pyrene and fluoranthene, are just as closely related (0·951), and the average correlation between pyrene and the other 3 substances is 0·923 compared with 0·929 for the more stable benzopyrene. It appears that the rate of change of pyrene in the samples must have been so uniform as not to affect correlations with other factors appreciably.

*Validity of correlations between smoke measurements and mortality rates*

A source of difficulty in attempts to correlate these factors is that neither is constant in time, and exposure to air pollution for a number of years may be necessary to produce serious respiratory disease or initiate cancer. In order to obtain meaningful rates from specific diseases in separate districts deaths through a period of years have to be aggregated and the rates standardized to allow for differing age distributions in the populations concerned. Changes in the international rules for classifying deaths to their underlying cause from 1950 onwards, and changes in the rules for residence allocation of persons dying from chronic diseases after a long time in hospital which took effect in 1954, made it advisable to use the period 1950–54 for lung cancer and 1950–53 for the other disease groups in this study. The smoke measurements were made for the most part during 1955–58. It has to be assumed, therefore, that although the amount and nature of air pollution by smoke may have changed in the last 10 or 20 years, the *relative* changes have been the same in all the areas concerned, in which case the correlations with other factors would not be affected. Departures from this assumed constancy of relative change would tend almost certainly to reduce the true correlations and the observed coefficients can safely be regarded as minimal figures. This difficulty must arise in all attempts to relate mortality from chronic diseases with measures of air pollution of which records exist only for recent years, and action which has now started to reduce such pollution in the worst areas makes it an urgent matter to make such epidemiological studies before the opportunity has gone.

Another possibly important source of error was the reliance on a single instrument site to measure the average air conditions to which the population of the district surrounding it was exposed in the course of a year. A study of this problem has been made in Sheffield, where it was found that when any 8 instruments were chosen to assess the average smoke concentration to which the whole of the population in the city was being exposed, the mean of the 8 had a coefficient of variation of about 10 per cent (Clifton *et al.*, 1959). This variation increased in the inverse ratio of the square root of the number of filter sites, reaching about 28 per cent for a single site. Sheffield is peculiar in having within its area of 62 square miles altitudes above sea level ranging from 100 to 1400 feet and densities of population in separate wards ranging from 2 to 62 per acre, and the coefficient of variation is undoubtedly higher than would be found in most cities. In Liverpool where the river is a disturbing factor, smoke concentrations at three locations were 179, 215 and 204 in the spring-summer half and 500, 624, 604 in autumn-winter, measured in mg./1000 $m^3$. In small towns variation would generally be less, and in the 26 areas used in the present study the coefficient of variation for a single site can reasonably be taken as 20 per cent.

In order to find what effect such errors could have on the correlation coefficients with mortality several tests were made on the lung cancer figures, assuming the root mean square error of the smoke readings to be 20 per cent of the true averages in the area. The 26 ratios (F) between the true average smoke value in a district and the smoke reading at a single location in it were presumed to have a normal distribution with standard deviation 0·20 and they were first arranged in order according to a table of random numbers and applied as correcting factors to the observed smoke figures in the areas when arranged in alphabetical order of place names. The resulting correlation coefficient between the corrected smoke values and lung cancer rates was 0·77, and when the factors in the same random order were applied to the areas arranged in order of their lung cancer rates the result was 0·82. It is to be expected, however, that the magnitude of the error, regardless of its sign, would in general increase with the size of the district providing the death rates, and the districts were therefore arranged in ascending order of their size and the presumed errors ($F - 1$) were also arranged by magnitude with + and − errors alternating, each smoke reading being then corrected by the corresponding factor ($F$) to estimate the true average for the district. This led to a correlation coefficient with lung cancer of 0·873, the same as the original uncorrected value (Table IV). The conclusion from these tests is that the errors arising from the use of only a single instrument site in each district have not affected the correlation coefficients sufficiently to invalidate the deductions made from such data in this paper. For greater accuracy it would be advisable in future stuides of this kind to instal, if practicable, more than one instrument in districts with population ($P$) exceeding 50,000. A convenient criterion for the number ($n$) to be used would be $n = \sqrt{P}/20{,}000$, the choice of sites being related to the population distribution within the area as suggested by the Sheffield study (Clifton *et al.*, 1959).

*Social environment*

Several measures of the average social conditions in administrative areas have been used by statisticians in the study of death rates. The most readly available one is the density of population per acre, and in international comparisons of towns this may be the only one which can be obtained from official data. Death rates from respiratory diseases are highly correlated with this measure of social conditions, but only in recent years has it been realised that the main reason for this is the simple fact that population per acre is in itself a measure, in Britain at least, of the number of domestic chimneys per acre and therefore of the average density of domestic smoke in the atmosphere of the area. There are other factors contributing to the correlations, for example districts with high densities of population tend to have more heavy industries, more unskilled workers and poorer housing conditions. That these other factors are of minor importance compared with smoke in producing the correlation of 0·839 in the 26 northern areas between lung cancer mortality of males and persons per acre (Table IV) is evident from the fact that this figure is reduced to 0·327 when smoke density is held constant. Population per acre is not, therefore, a good measure of non-atmospheric social conditions since it is so closely linked with smoke density that to hold it constant for 2nd order correlations over-corrects for the social factors. It was used in a previous study of towns (Stocks, 1959) only because at the time of preparing the paper no better index was available, but this erred on the safe side by reducing lung cancer and bronchitis correlations with smoke too severely.

Other social indices which have been used by the Registrar General in the past are the housing indices based on numbers of persons per room and the proportion of occupied and retired males in unskilled occupations. In the present paper two measures of the last mentioned kind have been used. After the census of 1931 the proportion of males aged 14 and over in social classes IV and V was employed as a " social index " for large towns (Registrar General, 1936), and that index has been used here in the study of smoke in county boroughs, and a similar index based on 1951 census has been used for 40 districts of Greater London (Tables XI, XIII and XV). Where rural areas or small country towns occur in the survey, social class IV may include large numbers of agricultural labourers whose mortality rates are much below the class average, and the proportion in class V alone is a better social index. For the 26 northern districts this latter index has been used (Tables III, IV, V, X), and also for the Lancashire and Yorkshire districts but in this case as an alternative the rural districts have been omitted and the index based on classes IV–V applied (Tables XII and XIV). In Greater London the correlations between smoke concentration and 4 social indices were found to be : persons per acre 0·776, persons per room 0·447, class IV–V index 0·382, class V index 0·368. In the Lancashire and Yorkshire districts the coefficient with persons per acre was 0·611, and when rural areas were omitted the coefficient with class IV–V index was 0·397.

*Smoke, polycyclic hydrocarbons and mortality in 26 areas*

In Table II the 26 filter locations which were chosen with a view to measuring average atmospheric conditions in the districts around them are arranged in order of the mean smoke concentrations during a complete year, ranging from 15 mg. per 1000 cubic metres of air in Conway Valley to 562 in Salford. The next 4 columns show the amounts of 3 : 4 benzopyrene, 1 : 12 benzoperylene, pyrene and fluoranthene in parts per million of the smoke, and the last 4 columns show the same hydrocarbons in micrograms per 1000 cubic metres of air. The hydrocarbon rates are based on the improved analytical methods introduced in 1958 and consequently some are not comparable with rates published previously, but all figures in the table are comparable one with another.

The geographical variation in amounts of the hydrocarbons per unit of smoke is less than that shown by the concentrations in air ; for the former the coefficients of variation of the 4 substances are 35, 39, 37 and 52, and for the latter they are 81, 72, 77 and 71. Irritant effects on the respiratory tract might depend on the number of particles of a particular size and containing a harmful substance, on the average amount of it present in a particle or simply on the total quantity of the substance inhaled regardless of its distribution in the particles. Since workers in very high concentrations of coal combustion products in gas works suffer only moderate irritant effects, the nature of the particles in domestic coal smoke must be important, but in comparing effects of ordinary atmospheres polluted by different amounts of domestic smoke it is to be expected that the particles collected by the standard filters will be similar in nature and that pathological effects will be most closely related to the total amounts of particular chemical substances inhaled, that is to say their concentration per unit volume of air. Table II shows that the annual average concentration of 3 : 4 benzopyrene ranged from 1 $\mu$g./1000 $m^3$ in the purest air to 108 in the most polluted ; for 1 : 12 benzoperylene it ranged from 0·4 to 74, for pyrene from 1 to 38 and for fluoranthene from 3 to 58.

Table III gives the population densities, social indices and standardised mortality ratios for cancer of the lung (males), bronchitis and pneumonia in the administrative areas where the 26 filters were located. Rates for stomach and breast cancer are not shown from considerations of space, but the S.M.R.'s for

TABLE II.—*Average Annual Amounts of Smoke and 4 Polycyclic Hydrocarbons in 26 Localities of Northern England and Wales,* 1957 *or* 1958.

| Location of filter (ranked according to smoke density) | Year | Smoke (mg./1000 cu. m.) | Hydrocarbons (µg. per g. of smoke) 3 : 4 Benzo-pyrene | 1 : 12 Benzo-perylene | Py-rene | Fluoran-thene | Hydrocarbons (µg. per 1000 cu. m. of air) 3 : 4 Benzo-pyrene | 1 : 12 Benzo-perylene | Py-rene | Fluoran-thene |
|---|---|---|---|---|---|---|---|---|---|---|
| Conway Valley | 7 | 15 | 75 | 37 | 81 | 263 | 0·9 | 0·4 | 0·8 | 3·3 |
| Llangefni | 7 | 53 | 198 | 82 | 118 | 169 | 6·8 | 3·1 | 5·0 | 4·6 |
| Tattenhall | 7 | 85 | 65 | 59 | 61 | 112 | 5·1 | 4·5 | 4·8 | 9·2 |
| Wetherby | 8 | 98 | 115 | 114 | 69 | 105 | 11·0 | 11·0 | 7·0 | 10·0 |
| Ruthin | 7 | 105 | 73 | 89 | 36 | 62 | 8·1 | 9·8 | 3·4 | 5·4 |
| Blaenau Ffestiniog | 7 | 124 | 69 | 95 | 81 | 187 | 5·5 | 21·0 | 12·0 | 20·0 |
| Ripon | 8 | 125 | 109 | 89 | 48 | 72 | 15·0 | 12·0 | 6·0 | 10·0 |
| Elland | 8 | 150 | 108 | 96 | 32 | 49 | 15·0 | 14·0 | 5·0 | 7·0 |
| Flint | 7 | 169 | 153 | 129 | 57 | 93 | 25·1 | 20·8 | 12·8 | 15·8 |
| York | 8 | 177 | 137 | 121 | 78 | 110 | 24·0 | 21·0 | 14·0 | 20·0 |
| Chester | 7 | 208 | 110 | 117 | 56 | 75 | 19·6 | 12·6 | 10·5 | 13·4 |
| Ormskirk | 7 | 226 | 123 | 136 | 45 | 66 | 26·1 | 28·0 | 10·7 | 16·1 |
| Wrexham | 7 | 264 | 128 | 96 | 60 | 60 | 24·9 | 24·7 | 12·0 | 15·2 |
| Lancaster | 8 | 276 | 71 | 49 | 34 | 51 | 20·0 | 14·0 | 9·0 | 14·0 |
| Darwen | 8 | 288 | 125 | 123 | 60 | 85 | 36·0 | 35·0 | 17·0 | 25·0 |
| Keighley | 8 | 291 | 109 | 108 | 34 | 61 | 32·0 | 32·0 | 10·0 | 18·0 |
| Burnley | 8 | 311 | 105 | 95 | 14 | 24 | 32·0 | 30·0 | 4·0 | 8·0 |
| Birkenhead | 7 | 336 | 169 | 153 | 71 | 89 | 47·1 | 42·3 | 20·7 | 23·7 |
| Bootle | 7 | 362 | 152 | 150 | 68 | 97 | 51·5 | 49·4 | 24·9 | 33·2 |
| St. Helens | 7 | 376 | 205 | 151 | 71 | 105 | 69·2 | 52·1 | 28·0 | 44·2 |
| Warrington | 7 | 380 | 123 | 98 | 34 | 64 | 48·8 | 41·0 | 14·3 | 28·3 |
| Gateshead | 8 | 399 | 156 | 125 | 73 | 101 | 62·0 | 50·0 | 29·0 | 40·0 |
| Leeds | 8 | 421 | 100 | 95 | 34 | 49 | 42·0 | 40·0 | 14·0 | 21·0 |
| Liverpool (Princes Rd.) | 7 | 455 | 241 | 182 | 66 | 108 | 99·9 | 74·2 | 29·8 | 47·9 |
| Newcastle-on-Tyne* | 8 | 505 | 149 | 145 | 74 | 115 | 75·0 | 73·0 | 37·0 | 58·0 |
| Salford (Regent Rd.) | 8 | 562 | 189 | 108 | 66 | 87 | 108·0 | 62·0 | 38·0 | 49·0 |
| Mean | — | 260 | 129·0 | 109·3 | 58·5 | 94·6 | 35·0 | 29·9 | 14·6 | 21·5 |

* Wharncliffe Street.

other cancer are given. The numbers of deaths of females from lung cancer in many of the areas were too small for reliable conclusions to be reached. All the S.M.R.'s are based on England and Wales as a whole taken as 100, and they range from 23 to 165 for lung cancer, 68 to 122 for other cancer, 18 to 259 for bronchitis and 61 to 227 for pneumonia.

A social factor which is related indirectly with density of population and directly with lung cancer incidence is the amount of cigarette smoking, and it is not possible to correct for this since complete data are lacking, but its importance in the North Wales and Merseyside region has been measured by actual enquiries into tobacco smoking histories of hospital patients. Lung cancer mortality in heavy smokers was found to differ little in urban and rural areas, in medium cigarette smokers the urban/rural ratio was about 1½ and in light smokers about 2½ (Stocks, 1958*a*). The effect of actually correcting for differences in smoking

TABLE III.—*Standardised Mortality Ratios for Cancer, Bronchitis and Pneumonia in 26 Areas of Northern England and Wales where Smoke Analyses have been made.*

| Administrative area and county‡ | | Persons per acre | Social class (V) index | S.M.R.'r in 1950–53* Lung cancer (M.) | Other sites of cancer† (M.) | Other sites of cancer† (F.) | Bronchitis (M.) | Bronchitis (F.) | Peumonia (M.) | Peumonia (F.) |
|---|---|---|---|---|---|---|---|---|---|---|
| Nant Conway R.D. | CA | 0·1 | 109 | 59 | 81 | 88 | 29 | 91 | 94 | 181 |
| Llangefni U.D. (1) | A | 0·2 | 105 | 54 | 110 | 104 | 18 | 48 | 126 | 100 |
| Tarvin R.D. (2) | CH | 0·2 | 107 | 59 | 68 | 97 | 55 | 57 | 61 | 76 |
| Wetherby R.D. | Y | 0·3 | 94 | 64 | 88 | 89 | 59 | 42 | 102 | 101 |
| Ruthin M.B. and R.D. | DE | 0·1 | 74 | 23 | 84 | 93 | 28 | 75 | 82 | 90 |
| Ffestiniog U.D. | M | 0·4 | 61 | 74 | 98 | 154 | 46 | 12 | 154 | 143 |
| Ripon U.D. and R.D. | Y | 0·2 | 149 | 72 | 84 | 93 | 66 | 88 | 146 | 107 |
| Elland U.D. | Y | 3·2 | 99 | 70 | 81 | 106 | 79 | 59 | 114 | 123 |
| Flint M.B. | F | 2·1 | 238 | 80 | 112 | 144 | 50 | 105 | 118 | 40 |
| York C.B. | Y | 16·4 | 162 | 92 | 113 | 106 | 95 | 75 | 120 | 113 |
| Chester C.B. | CH | 11·6 | 156 | 115 | 98 | 97 | 113 | 81 | 93 | 102 |
| Ormskirk U.D. | L | 1·3 | 142 | 92 | 92 | 110 | 82 | 97 | 109 | 86 |
| Wrexham M.B. | DE | 10·6 | 141 | 76 | 118 | 99 | 82 | 68 | 125 | 90 |
| Lancaster M.B. | L | 5·0 | 199 | 95 | 91 | 108 | 81 | 76 | 117 | 93 |
| Darwen U.D. | L | 5·2 | 211 | 81 | 104 | 109 | 112 | 136 | 91 | 125 |
| Keighley M.B. | Y | 2·4 | 116 | 76 | 104 | 119 | 116 | 167 | 91 | 92 |
| Burnley C.B. | L | 18·1 | 156 | 92 | 90 | 99 | 139 | 181 | 134 | 117 |
| Birkenhead C.B. | CH | 16·6 | 222 | 137 | 117 | 103 | 125 | 116 | 180 | 141 |
| Bootle C.B. | L | 13·1 | 295 | 143 | 85 | 92 | 194 | 164 | 214 | 219 |
| St. Helens C·B. | L | 13·9 | 227 | 111 | 102 | 109 | 160 | 141 | 144 | 165 |
| Warrington C.B. | L | 18·3 | 246 | 125 | 118 | 96 | 199 | 223 | 132 | 154 |
| Gateshead C.B. | DU | 25·7 | 192 | 113 | 113 | 109 | 136 | 111 | 164 | 119 |
| Leeds C.B. | Y | 13·2 | 135 | 134 | 104 | 99 | 162 | 141 | 200 | 202 |
| Liverpool C.B. | L | 28·9 | 245 | 160 | 119 | 103 | 139 | 138 | 227 | 245 |
| Newcastle-on-Tyne | N | 26·3 | 166 | 123 | 118 | 103 | 114 | 113 | 161 | 126 |
| Salford C.B. | L | 34·2 | 209 | 165 | 122 | 112 | 259 | 240 | 176 | 154 |

*Notes*

(1) With Aethwy and Twrcelyn R.D's. (2) District surrounding Tattenhall. * 1950–54 for lung cancer. † All sites except lung, stomach, breast. ‡ A, Anglesey ; CA, Caernarvon ; CH, Cheshire ; DE, Denbigh ; DU, Durham ; F, Flint ; L, Lancashire ; M, Merioneth ; N, Northumberland; Y, Yorkshire West Riding.

habits between North Wales and Liverpool was to reduce the urban/rural ratio for lung cancer rates in men from 2·4 observed to 2·1, and for smaller towns the effect was less than this (Stocks, 1955). A sampling enquiry by the Tobacco Manufacturers' Standing Committee (1959) showed that the average daily consumpton of packeted cigarettes per adult in 1956 was 9 in rural districts, 9·6 in London and about 11 in other large towns. Applying these to the regression graphs of lung cancer mortality on number of cigarettes smoked in the same kind of area, the rate in large towns might be enhanced by about 20 per cent because of this difference. As a test of the effect this could have on the correlation between lung cancer rates and smoke density the S.M.R.'s in Table III were reduced to that extent for the 8 towns of over 100,000 population and the resulting coefficient was 0·72 instead of 0·87, the uncorrected figure. The coefficients of 0·52 in 30 county boroughs (Table XIII) and 0·53 in 42 Lancashire and Yorkshire urban areas (Table XIV) are unlikely to be affected appreciably by differences in smoking habits.

TABLE IV.—*Correlations in 26 Localities of Northern England and Wales between Smoke and Hydrocarbons in Air and Standardised Mortality from Lung Cancer in Males in* 1950–54, *Population Density and Social* (*Class V*) *Index.*

| | 1st order coefficients with | | | | 2nd order with lung cancer, with constant | | |
|---|---|---|---|---|---|---|---|
| | Lung cancer | Smoke | Persons per acre | Social index | P.P.A. | Index | Smoke |
| | Smoke and hydrocarbons per cubic metre of air | | | | | | |
| Smoke (total) | 0·873 | — | 0·871 | 0·649 | 0·531 | 0·756 | — |
| 3 : 4 Benzopyrene | 0·862 | 0·927 | 0·895 | 0·662 | 0·457 | 0·738 | 0·288 |
| 1 : 12 Benzoperylene | 0·832 | 0·936 | 0·856 | 0·667 | 0·409 | 0·667 | 0·090 |
| Pyrene | 0·751 | 0·848 | 0·798 | 0·635 | 0·250 | 0·543 | 0·045 |
| Fluoranthene | 0·779 | 0·876 | 0·821 | 0·619 | 0·293 | 0·622 | 0·020 |
| Lung cancer | — | — | 0·839 | 0·735 | — | — | — |
| | Hydrocarbons per unit weight of suspended matter | | | | | | |
| 3 : 4 Benzopyrene | 0·587 | — | 0·604 | 0·591 | 0·185 | 0·272 | — |
| 1 : 12 Benzoperylene | 0·464 | — | 0·411 | 0·606 | 0·240 | 0·044 | — |
| Pyrene | 0·004 | — | 0·073 | −0·084 | −0·109 | 0·096 | — |
| Fluoranthene | −0·180 | — | −0·248 | −0·252 | 0·052 | 0·018 | — |

*Correlations between polycyclic hydrocarbons and lung cancer rates*

Table IV shows the correlaton coefficients between lung cancer mortality in men and the social and air-pollution indices, and partial coefficients when the social factors are constant. For the reasons already stated elimination of persons per acre reduces the primary coefficients unduly, but the figures are given for comparison with those corrected for proportions of men in social class V which are free from that objection. Concentrations of the hydrocarbons in air are highly correlated with lung cancer after eliminating the latter social factor, with odds exceeding 1000 to 1 against such coefficients arising fortuitously in the case of 3 of them. All coefficients exceeding 0·375 are significant at the conventional level of 1 in 20 probability. Amounts of the substances per unit of smoke show no significant associations with lung cancer, and only in the case of 3 : 4 benzopyrene is there any suggestion that this may be of any importance.

Table VI indicates in its first section that the hydrocarbon concentrations in air are very highly correlated one with another, owing to their common origin, and when a substance $x$ is present in large amount the others must tend to be increased also in consequence. If $x$ is concerned in disease incidence and the others are not, the correlations between the others and the disease rates should become inappreciable when the concentration of $x$ is held constant. In effect this means that in 26 areas having the same degree of air pollution by the active substance $x$ no correlations would be found between lung cancer mortality and the amounts of the other hydrocarbons present in the air. In the first column of the second section of the table the concentration of each hydrocarbon has been held constant in turn, with the following result for lung cancer. With 3 : 4 benzopyrene constant the total smoke coefficient is reduced from 0·873 (Table IV) to

0·389, indicating the probable presence of some other constituents of smoke which affect the lung, such as certain trace elements as will be seen later ; the benzoperylene coefficient is reduced to 0·055, and the other two hydrocarbons cease to show any relation with lung cancer. With 1 : 12 benzoperylene held constant the 3 : 4 benzopyrene coefficient is still significant (0·394) whereas the others disappear; and the making of pyrene and fluoranthene constant scarcely affects the benzopyrene coefficient and leaves the benzoperylene figure still significant. As the figures at the foot of the first column indicate, pyrene and fluoranthene derive their statistical connections with lung cancer from their association with 3 : 4 benzopyrene in smoke ; 1 : 12 benzoperylene may have an independent effect, but if so it is small in comparison with that of 3 : 4 benzopyrene which clearly emerges from this analysis as the hydrocarbon of outstanding importance. These results agree with experimental evidence about the carcinogenic potency of the hydrocarbons.

*Correlations between polycyclic hydrocarbons and mortality from other diseases*

Table V shows no appreciable correlations between amounts of smoke or any of the hydrocarbons in air and mortality from cancer of the stomach in either sex or cancer of the breast or other organs in females after correcting for social index. Other cancer in males, for which figures are not shown in the tables, gives primary coefficients of 0·64 with smoke, 3 : 4 benzopyrene and pyrene, 0·63 with 1 : 12 benzoperylene and 0·61 with fluoranthene, and when social index and other hydrocarbons are constant the benzopyrene and pyrene coefficients are still appreciable but not significant and no conclusion can be drawn. The group includes the larynx, oesophagus and intestine, of which separate data are not obtainable.

Bronchitis gives primary correlations similar to those for lung cancer, and the partial coefficients when social index is constant are significant for males in respect of each hydrocarbon and for females in respect of 3 : 4 benzopyrene and

TABLE V.—*Correlations in 26 Localities of Northern England and Wales between Smoke and Hydrocarbons in Air, Standardised Mortality from Cancer of the Stomach, Breast and Other Sites, Bronchitis and Pneumonia in* 1950–53, *and Social (Class V) Index.*

| | Cancer (excluding lung) | | | | Bronchitis | | Pneumonia | |
|---|---|---|---|---|---|---|---|---|
| | Stomach | | Breast | Other | | | | |
| | (M.) | (F.) | (F.) | (F.) | (M.) | (F.) | (M.) | (F.) |
| | | | | 1st order coefficients | | | | |
| Smoke (total) | 0·241 | 0·308 | 0·104 | −0·002 | 0·869 | 0·751 | 0·666 | 0·479 |
| 3 : 4 Benzopyrene | 0·261 | 0·305 | 0·136 | 0·024 | 0·809 | 0·699 | 0·699 | 0·549 |
| 1 : 12 Benzoperylene | 0·141 | 0·281 | 0·129 | 0·013 | 0·741 | 0·672 | 0·715 | 0·511 |
| Pyrene | 0·045 | 0·219 | 0·071 | 0·093 | 0·683 | 0·530 | 0·662 | 0·443 |
| Fluoranthene | 0·118 | 0·319 | −0·022 | 0·101 | 0·700 | 0·451 | 0·636 | 0·583 |
| | | | 2nd order coefficients with social index constant | | | | | |
| Smoke (total) | 0·123 | 0·077 | — | — | 0·768 | 0·570 | 0·491 | 0·256 |
| 3 : 4 Benzopyrene | 0·146 | 0·073 | — | — | 0·693 | 0·513 | 0·540 | 0·356 |
| 1 : 12 Benzoperylene | *Nil* | 0·048 | — | — | 0·532 | 0·430 | 0·565 | 0·297 |
| Pyrene | ,, | 0·077 | — | — | 0·448 | 0·211 | 0·490 | 0·209 |
| Fluoranthene | ,, | 0·140 | — | — | 0·489 | 0·094 | 0·455 | 0·418 |

1 : 12 benzoperylene. Table VI shows however that when 3 : 4 benzopyrene is held constant the other hydrocarbons cease to have any association with bronchitis in either sex, whereas the benzopyrene coefficients average 0·584 when the others are held constant. This points to 3 : 4 benzopyrene as the hydrocarbon concerned with bronchitis mortality.

Pneumonia shows in Table V significant correlations when social index is constant for males in respect of each hydrocarbon and for females in respect of

TABLE VI.—*Correlations with Amounts of Smoke and Hydrocarbons in Air of 26 Localities when the Effect of One Hydrocarbon on the Coefficient is Eliminated.*

*Inter-correlations between Smoke and Hydrocarbons*

| | Smoke | Benzopyrene | Benzo-perylene | Pyrene | Fluoran-thene |
|---|---|---|---|---|---|
| 3 : 4 Benzopyrene | 0·927 | — | 0·956 | 0·915 | 0·917 |
| 1 : 12 Benzoperylene | 0·936 | 0·956 | — | 0·902 | 0·927 |
| Pyrene | 0·848 | 0·915 | 0·902 | — | 0·951 |
| Fluoranthene | 0·876 | 0·917 | 0·927 | 0·951 | — |

*Correlations with Indices of Mortality*

| | Cancer of lung | Bronchitis | | Pneumonia | |
|---|---|---|---|---|---|
| | (M.) | (M.) | (F.) | (M.) | (F.) |
| | 2nd order coefficients with one hydrocarbon constant | | | | |
| *Benzopyrene constant* | | | | | |
| Smoke (total) | 0·389 | 0·538 | 0·386 | 0·068 | *Nil* |
| 1: 12 Benzoperylene | 0·055 | *Nil* | 0·017 | 0·223 | ,, |
| Pyrene | *Nil* | ,, | *Nil* | 0·081 | ,, |
| Fluoranthene | ,, | ,, | ,, | *Nil* | 0·240 |
| *Benzoperylene constant* | | | | | |
| Smoke (total) | 0·480 | 0·741 | 0·470 | ,, | 0·002 |
| 3 : 4 Benzopyrene | 0·394 | 0·511 | 0·259 | 0·073 | 0·237 |
| Pyrene | 0·002 | 0·040 | *Nil* | 0·058 | *Nil* |
| Fluoranthene | 0·065 | 0·077 | ,, | *Nil* | 0·333 |
| *Pyrene constant* | | | | | |
| Smoke (total) | 0·674 | 0·748 | 0·671 | 0·262 | 0·173 |
| 3 : 4 Benzopyrene | 0·654 | 0·625 | 0·624 | 0·306 | 0·443 |
| 1 : 12 Benzoperylene | 0·542 | 0·397 | 0·535 | 0·363 | 0·288 |
| Fluoranthene | 0·318 | 0·225 | *Nil* | 0·026 | 0·581 |
| *Fluoranthene constant* | | | | | |
| Smoke (total) | 0·629 | 0·741 | 0·828 | 0·293 | *Nil* |
| 3 : 4 Benzopyrene | 0·589 | 0·586 | 0·899 | 0·376 | 0·043 |
| 1 : 12 Benzoperylene | 0·468 | 0·344 | 0·760 | 0·434 | *Nil* |
| Pyrene | 0·055 | 0·058 | 0·366 | 0·242 | ,, |
| | Per cent reduction of smoke coefficients by eliminating one hydrocarbon | | | | |
| *Hydrocarbon constant* | | | | | |
| 3 : 4 Benzopyrene | 56 | 38 | 49 | 90 | 100 |
| 1 : 12 Benzoperylene | 46 | 15 | 37 | 100 | 100 |
| Pyrene | 25 | 14 | 11 | 61 | 64 |
| Fluoranthene | 30 | 15 | 0 | 56 | 100 |
| | Per cent reduction of other hydrocarbon correlations by eliminating 3 : 4 benzopyrene | | | | |
| *Benzopyrene constant* | | | | | |
| 1 : 12 Benzoperylene | 93 | 100 | 97 | 69 | 100 |
| Pyrene | 100 | 100 | 100 | 88 | 100 |
| Fluoranthene | 100 | 100 | 100 | 100 | 59 |

fluoranthene, but in Table VI the results of eliminating the substances individually are different from those for lung cancer and bronchitis. When 1 : 12 benzoperylene is made constant all the other correlations disappear for males whilst fluoranthene remains predominant for females, and this suggests that these may be concerned with the incidence of some cases of pneumonia whereas benzopyrene is less important.

*Trace elements and mortality in 23 areas*

The amounts of 13 elements in the smoke samples were determined spectrographically at the Fuel Research Station of the Department of Scientific and Industrial Research by Mr. K. V. Aubrey. Filter papers were changed at intervals of a week or longer and the suspended matter collected throughout a whole year was aggregated for analysis. In 1956 when 10 stations were operating about 500 cubic feet of air per week passed through a standard 2-inch or a special 4-inch diameter filter according to the degree of air-pollution anticipated ; but in 1957 and 1958 when more stations were introduced about 3000 cubic feet per week were drawn through a 4-inch filter to obtain larger quantities of smoke for analysis. This was done at 23 of the 26 representative locations in Table I, and the results are given in Tables VII*a* and VII*b*. Analyses of copper and chromium are available for 14 of these.

TABLE VII*a*.—*Average Annual Amounts of Total Ash, Lead, Zinc, Copper, Titanium, Arsenic and Manganese per* 1000 *cubic metres of Air at* 23 *Localities in Northern England and Wales in* 1956–58.

| | | mg. | | μg. | | | | |
|---|---|---|---|---|---|---|---|---|
| Locality | Year | Total ash | Lead | Zinc | Copper | Titanium | Arsenic | Manganese |
| Conway Valley | 1957 | 4·0 | 0·08 | 48 | 7 | 11 | 5 | 5 |
| Llangefni | 1956 | 6·0 | 0·27 | 76 | — | 10 | 11 | 10 |
| Tattenhall | 1956 | 8·0 | 0·22 | 130 | — | 30 | 23 | 27 |
| Wetherby | 1958 | 6·6 | 0·35 | 205 | 38 | 17 | 27 | 13 |
| Ruthin | 1957 | 8·5 | 0·47 | 73 | 14 | 33 | 13 | 10 |
| Ripon | 1958 | 6·5 | 0·40 | 100 | 21 | 15 | 23 | 13 |
| Elland | 1958 | 7·2 | 0·42 | 285 | 50 | 16 | 32 | 12 |
| Flint | 1956 | 12·5 | 0·29 | 230 | — | 43 | 33 | 40 |
| York | 1958 | 11·4 | 0·66 | 295 | 45 | 30 | 59 | 23 |
| Chester | 1956 | 12·0 | 0·50 | 250 | — | 30 | 46 | 39 |
| Ormskirk | 1957 | 10·0 | 0·52 | 225 | 45 | 25 | 35 | 16 |
| Wrexham | 1956 | 10·0 | 0·39 | 250 | — | 10 | 42 | 35 |
| Lancaster | 1958 | 8·9 | 0·63 | 185 | 33 | 23 | 39 | 12 |
| Darwen | 1958 | 11·6 | 0·71 | 185 | 32 | 36 | 37 | 52 |
| Keighley | 1958 | 13·0 | 0·71 | 140 | 44 | 42 | 41 | 43 |
| Burnley | 1958 | 16·1 | 0·95 | 185 | 34 | 71 | 44 | 32 |
| Birkenhead | 1956 | 13·0 | 0·60 | 240 | — | 20 | 68 | 35 |
| Bootle | 1956 | 22·0 | 0·58 | 490 | — | 50 | 130 | 50 |
| St. Helens | 1956 | 15·0 | 0·44 | 330 | — | 40 | 160 | 32 |
| Warrington | 1957 | 24·0 | 0·75 | 440 | 53 | 84 | 51 | 50 |
| Leeds | 1958 | 37·1 | 1·30 | 370 | 96 | 180 | 60 | 130 |
| Liverpool (Princes Rd.) | 1956 | 15·0 | 0·57 | 280 | — | 60 | 94 | 34 |
| Salford (Regent Rd.) | 1958 | 21·0 | 1·43 | 335 | 252 | 75 | 74 | 43 |

*Note*.—The localities are ranked in order of smoke density as in Table II.

TABLE VII*b*.—*Average Annual Amounts of Nickel, Antimony, Vanadium, Chromium, Molybdenum, Cobalt and Beryllium per* 1000 *cubic metres of Air at* 23 *Localities in Northern England and Wales in* 1956–58.

| Locality | Year | Nickel (μg.) | Antimony | Vanadium | Chromium | Molybdenum | Cobalt | Beryllium |
|---|---|---|---|---|---|---|---|---|
| Conway Valley | 1957 | 1·3 | 0·9 | 1·2 | 0·9 | 0·29 | 0·25 | 0·06 |
| Llangefni | 1956 | 2·3 | 4·0 | 3·4 | — | 0·20 | 0·70 | 0·20 |
| Tattenhall | 1956 | 4·1 | 5·3 | 6·9 | — | 0·40 | 1·30 | 0·20 |
| Wetherby | 1958 | 74·5 | 5·3 | 1·6 | 2·7 | 1·30 | 0·85 | 0·14 |
| Ruthin | 1957 | 1·8 | 3·3 | 1·2 | 1·6 | 0·60 | 0·74 | 0·15 |
| Ripon | 1958 | 31·2 | 4·5 | 1·5 | 2·9 | 1·19 | 0·83 | 0·14 |
| Elland | 1958 | 205·0 | 8·6 | 1·1 | 2·1 | 1·25 | 2·30 | 0·19 |
| Flint | 1956 | 6·9 | 7·0 | 5·9 | — | 1·00 | 1·20 | 0·50 |
| York | 1958 | 29·0 | 7·7 | 5·1 | 5·4 | 2·35 | 1·13 | 0·27 |
| Chester | 1956 | 12·0 | 12·0 | 7·6 | — | 1·90 | 1·60 | 0·60 |
| Ormskirk | 1957 | 16·0 | 12·5 | 4·6 | 2·7 | 1·40 | 0·97 | 0·26 |
| Wrexham | 1956 | 6·9 | 8·8 | 4·6 | — | 1·40 | 1·30 | 0·60 |
| Lancaster | 1958 | 17·3 | 7·2 | 5·4 | 2·3 | 1·56 | 0·78 | 0·21 |
| Darwen | 1958 | 11·2 | 9·5 | 8·8 | 4·0 | 3·40 | 1·05 | 0·31 |
| Keighley | 1958 | 32·5 | 13·0 | 3·7 | 4·1 | 2·50 | 2·05 | 0·31 |
| Burnley | 1958 | 13·5 | 8·6 | 6·8 | 4·8 | 4·45 | 1·55 | 0·40 |
| Birkenhead | 1956 | 10·0 | 12·0 | 31·0 | — | 2·00 | 1·60 | 0·60 |
| Bootle | 1956 | 23·0 | 20·0 | 42·0 | — | 1·80 | 2·00 | 1·00 |
| St. Helens | 1956 | 24·0 | 250·0 | 18·0 | — | 3·20 | 1·60 | 0·90 |
| Warrington | 1957 | 37·0 | 21·5 | 12·0 | 4·4 | 5·00 | 2·90 | 0·81 |
| Leeds | 1958 | 23·0 | 16·0 | 15·0 | 21·5 | 6·10 | 4·25 | 0·84 |
| Liverpool (Princes Rd.) | 1956 | 11·0 | 16·0 | 30·0 | — | 2·70 | 1·40 | 1·00 |
| Salford (Regent Rd.) | 1958 | 32·0 | 18·5 | 14·2 | 7·0 | 5·60 | 2·60 | 0·67 |

*Note*.—The localities are ranked in order of smoke density as in Table II.

For a few of the elements one or two places showed concentrations outside the normal range, as for example copper at Salford, titanium and manganese at Leeds, nickel at Elland, antimony at St. Helens, the presumed reason being proximity of special industries, but for the most part the distributions approximated to the normal type. The total ash ranged from 4 to 37 mg. per 1000 cubic metres of air ; in Birkenhead, Liverpool, St. Helens and Salford it formed about 4 per cent of the total smoke, and in country districts about 7 to 10 per cent. The concentrations of 11 elements were correlated with the standardized mortality ratios in Table III with results shown in Tables VIII–X.

*Correlations between trace elements and lung cancer*

Table VIII shows that for each element except nickel and antimony concentration in air is correlated significantly with lung cancer mortality, the critical value at 1 in 20 level of significance being 0·40. Table IX reveals the effect on the total smoke coefficient (0·860) of making the amount of one element constant, and only for the first 5 elements, beryllium, arsenic, zinc, molybdenum and vanadium is this appreciable. The lower part of Table X shows that when the beryllium concentration is held constant the molybdenum correlation remains significant ; and when molybdenum is constant the beryllium and vanadium

TABLE VIII.—*Correlations in* 23 *Localities of Northern England and Wales between Amounts of Trace Elements in Air, Standardised Mortality from Cancer, Bronchitis and Pneumonia in* 1950–53 *and Proportion of Males aged* 15 *and over in Social Class V in the Population.*

1st order coefficients

| | Cancer of Lung (M.) | Cancer of Breast (F.) | Other cancer except stomach (M.) | Other cancer except stomach (F.) | Ash (total) |
|---|---|---|---|---|---|
| Beryllium | 0·827 | 0·129 | 0·550 | 0·124 | 0·729 |
| Arsenic | 0·748 | −0·080 | 0·321 | 0·086 | 0·534 |
| Zinc | 0·759 | 0·165 | 0·405 | 0·054 | 0·765 |
| Molybdenum | 0·684 | 0·028 | 0·520 | 0·109 | 0·746 |
| Vanadium | 0·770 | 0·053 | 0·556 | −0·061 | 0·513 |
| Cobalt | 0·536 | 0·220 | 0·296 | 0·063 | 0·692 |
| Nickel | −0·095 | 0·112 | −0·241 | −0·110 | 0·022 |
| Manganese | 0·521 | 0·351 | 0·400 | 0·162 | 0·928 |
| Lead | 0·626 | −0·017 | 0·462 | 0·149 | 0·908 |
| Titanium | 0·527 | 0·082 | 0·338 | 0·106 | 0·936 |
| Antimony | 0·327 | 0·132 | 0·230 | 0·148 | 0·261 |
| Social index | 0·724 | 0·149 | 0·452 | 0·266 | 0·452 |

| | Bronchitis (M.) | Bronchitis (F.) | Pneumonia (M.) | Pneumonia (F.) | Social index |
|---|---|---|---|---|---|
| Beryllium | 0·767 | 0·642 | 0·760 | 0·783 | 0·670 |
| Arsenic | 0·743 | 0·513 | 0·684 | 0·643 | 0·745 |
| Zinc | 0·796 | 0·547 | 0·615 | 0·674 | 0·676 |
| Molybdenum | 0·842 | 0·816 | 0·482 | 0·447 | 0·399 |
| Vanadium | 0·620 | 0·463 | 0·805 | 0·711 | 0·763 |
| Cobalt | 0·710 | 0·599 | 0·498 | 0·497 | 0·215 |
| Nickel | 0·095 | −0·015 | −0·053 | 0·020 | −0·232 |
| Manganese | 0·556 | 0·473 | 0·310 | 0·190 | 0·289 |
| Lead | 0·778 | 0·722 | 0·489 | 0·334 | 0·315 |
| Titanium | 0·602 | 0·597 | 0·495 | 0·512 | 0·223 |
| Antimony | 0·380 | 0·239 | 0·132 | 0·333 | 0·211 |
| Social index | 0·649 | 0·598 | 0·639 | 0·447 | — |

coefficients exceed 0·53 and the arsenic and zinc coefficients remain appreciable though not significant at the conventional level. Both beryllium and molybdenum appear to be associated with lung cancer incidence, and a suitably weighted combination of the two elements produces a high correlation of 0·720 with mortality after eliminating social index.

The connection with beryllium is supported by some experimental evidence of bronchogenic cancers in rats after inhaling beryllium dust for a long period, and several cases of lung cancer in men following berylliosis have been reported. Beryllium dust is known to be a respiratory irritant and many hundreds of cases of chronic berylliosis have occurred in processing the metal from ores (Hueper, 1957). It is of considerable interest to now find epidemiological indications of a connection between the small amounts of this element in ordinory air and the incidence of lung cancer amongst men who are continually inhaling it. Experimental evidence for any carcinogenic effects of molybdenum seems to be lacking, but this may be because it has not been sought for; the very high correlations noted below with bronchitis in both sexes are remarkable and the element might well be concerned also in lung cancer incidence.

Arsenic is correlated significantly (0·427) with lung cancer after eliminating the beryllium and molybdenum, and this tends to strengthen a view, long held, that this element in oxide form can have carcinogenic effects on the lung. Zinc gives a coefficient not quite significant at 1 in 20 level (0·379) and this might be meaningful in view of the evidence for a connection between its concentration in soil and the incidence of cancer of the stomach (Stocks and Davies, 1960). Vanadium also retains an appreciable correlation (0·347) after eliminating beryllium and molybdenum, and since it is a respiratory irritant some attention might be paid to it along with molybdenum as possible carcinogenic agents despite the present absence of clinical or experimental evidence.

*Correlations between trace elements and other diseases*

Table VIII shows no significant correlations between mortality from breast cancer and concentration of any trace element. The coefficient with manganese is reduced to 0·325 when social index is constant. Other cancer (excluding stomach) in females likewise shows no appreciable association with amount of smoke or of any element. In males however there is a smoke correlation of 0·603, which is reduced considerably by separate elimination of beryllium, molybdenum and vanadium. When social index and molybdenum concentration are

TABLE IX.—*Correlations between Mortality and Amount of Smoke in Air of 23 Localities when the Effect of one Trace Element on the Coefficient is Eliminated.*

| | Lung cancer (M.) | Other cancer* (M.) | Bronchitis (M.) | Bronchitis (F.) | Pneumonia (M.) | Pneumonia (F.) |
|---|---|---|---|---|---|---|
| | 1st order coefficients with smoke | | | | | |
| Smoke | 0·860 | 0·603 | 0·919 | 0·843 | 0·712 | 0·564 |
| *Constant factor* | 2nd order coefficients with smoke, one element constant | | | | | |
| Beryllium | 0·479 | 0·321 | 0·791 | 0·498 | 0·242 | *Nil* |
| Arsenic | 0·712 | 0·570 | 0·822 | 0·801 | 0·425 | 0·179 |
| Zinc | 0·733 | 0·489 | 0·847 | 0·768 | 0·504 | 0·189 |
| Molybdenum | 0·740 | 0·360 | 0·735 | 0·521 | 0·634 | 0·385 |
| Vanadium | 0·767 | 0·307 | 0·846 | 0·785 | 0·430 | 0·273 |
| Cobalt | 0·811 | 0·692 | 0·888 | 0·752 | 0·592 | 0·377 |
| Nickel | 0·880 | 0·602 | 0·936 | 0·848 | 0·711 | 0·568 |
| Manganese | 0·815 | 0·498 | 0·881 | 0·793 | 0·685 | 0·552 |
| Lead | 0·872 | 0·474 | 0·787 | 0·630 | 0·686 | 0·632 |
| Titanium | 0·812 | 0·535 | 0·871 | 0·753 | 0·609 | 0·366 |
| Antimony | 0·842 | 0·573 | 0·905 | 0·838 | 0·723 | 0·501 |
| | Per cent reduction of smoke coefficients by eliminating one trace element | | | | | |
| Beryllium | 44 | 47 | 14 | 41 | 66 | 100 |
| Arsenic | 17 | 9 | 10 | 5 | 40 | 68 |
| Zinc | 15 | 19 | 8 | 9 | 29 | 66 |
| Molybdenum | 14 | 40 | 8 | 38 | 11 | 32 |
| Vanadium | 11 | 51 | 8 | 7 | 40 | 55 |
| Cobalt | 6 | 0 | 3 | 11 | 17 | 33 |
| Nickel | 0 | 0 | 0 | 0 | 0 | 0 |
| Manganese | 5 | 21 | 4 | 6 | 4 | 2 |
| Lead | 0 | 17 | 14 | 25 | 4 | 0 |
| Titanium | 6 | 11 | 5 | 11 | 14 | 35 |
| Antimony | 2 | 5 | 1 | 0 | 0 | 11 |

* Excluding stomach.

held constant the correlations of other cancer with zinc, manganese and lead disappear but the coefficient with vanadium remains significant (0·396). This suggests that some forms of male cancer as well as lung may be affected by beryllium, molybdenum and vanadium.

Bronchitis shows in Table VIII correlations similar to those of lung cancer, and in the case of females the high coefficient with smoke is seen from Table IX to be reduced considerably by eliminating beryllium, molybdenum and lead. When social index and molybdenum are held constant the correlations with beryllium and lead virtually disappear, but molybdenum retains a high coefficient of 0·706 with female bronchitis after eliminating social index and beryllium. Bronchitis in males shows relations with the elements similar to those of lung cancer. With social index and beryllium constant the correlation with molybdenum is 0·712 ; when social index and molybdenum are eliminated arsenic and vanadium give coefficients of 0·563 and 0·469, beryllium gives 0·366 and zinc 0·360 ; and when social index and arsenic are constant molybdenum gives 0·850, beryllium 0·360 and zinc 0·425. It appears that molybdenum in the air is con-

TABLE X.—*Correlations in 23 Localities between Concentrations of Trace Elements in Air and Mortality when the Social Index and Other Trace Elements are Held Constant.*

2nd order inter-correlations between trace elements when social index is constant

| | Molybdenum | Arsenic | Zinc | Vanadium | Cobalt | Manganese | Lead |
|---|---|---|---|---|---|---|---|
| Beryllium | 0·495 | 0·756 | 0·652 | 0·610 | 0·552 | — | 0·311 |
| Molybdenum | — | 0·293 | 0·492 | *Nil* | 0·771 | 0·670 | 0·849 |
| *Both together* | — | 0·224 | 0·406 | 0·259 | 0·719 | — | — |
| Vanadium | *Nil* | 0·496 | — | — | 0·168 | — | — |
| Zinc | — | 0·541 | — | — | — | — | — |

3rd order coefficients with lung cancer, eliminating social index and one or more trace elements

| *Element constant* | Beryllium | Molybdenum | Arsenic | Zinc | Vanadium | Cobalt | Lead |
|---|---|---|---|---|---|---|---|
| Beryllium | — | 0·451 | — | — | — | 0·307 | — |
| Molybdenum | 0·552 | — | 0·389 | 0·337 | 0·538 | — | — |
| *Both together* | — | — | 0·427 | 0·379 | 0·347 | 0·096 | — |
| Arsenic | 0·587 | 0·591 | — | — | — | — | — |
| Similar coefficients with other diseases<br>Bronchitis (males) | | | | | | | |
| Beryllium | — | 0·712 | — | — | — | — | — |
| Molybdenum | 0·366 | — | 0·563 | 0·360 | 0·469 | 0·296 | 0·294 |
| Arsenic | 0·360 | 0·850 | — | 0·425 | — | — | — |
| Bronchitis (females) | | | | | | | |
| Beryllium | — | 0·706 | — | — | — | — | — |
| Molybdenum | 0·046 | — | — | — | — | — | 0·144 |
| Pneumonia (males) | | | | | | | |
| Beryllium | — | 0·048 | *Nil* | *Nil* | 0·443 | 0·236 | — |
| Vanadium | 0·316 | 0·416 | 0·136 | — | — | — | — |
| Pneumonia (females) | | | | | | | |
| Beryllium | — | *Nil* | *Nil* | 0·174 | *Nil* | 0·268 | — |

nected with bronchitis mortality in both sexes, and that arsenic, vanadium, beryllium and zinc may be concerned also in the case of males. The effects of beryllium on the respiratory tract have been referred to in reference to lung cancer. Vanadium pentoxide dust has for long been known to be an irritant to the upper respiratory tract, sometimes producing bronchitis or pneumonia amongst men working with it (Wyers, 1946 ; Sjoberg, 1950, 1956).

Pneumonia shows strongest primary correlations with beryllium, vanadium, arsenic and zinc, and the coefficients with smoke, which are not so great as for bronchitis and lung cancer, are reduced considerably by eliminating any of these elements. When social index and beryllium are held constant a significant coefficient remains only for vanadium (0·443) in the case of males, and all correlations disappear or become insignificant for females. It appears that beryllium is important for pneumonia in both sexes, and vanadium also in males.

*Association of standardised mortality from cancer, bronchitis and pneumonia with amounts of smoke and atmospheric deposit in urban areas of England and Wales*

In a previous paper (Stocks, 1959) correlations for cancer and bronchitis were given in respect of smoke collected by filters and undissolved deposit of larger particles in the county boroughs of England and Wales and districts of Lancashire and the West Riding of Yorkshire where data were available for one or more years from the reports of the D.S.I.R. The present study extends that analysis in the following ways : (1) records have become available for some new areas (2) cancer of the lung has been examined for each sex and cancer of the oesophagus distinguished where possible, (3) pneumonia has been added, (4) a similar study has been made of all the parts of Greater London for which data are available, (5) social conditions have been assessed by proportions of men in unskilled and partly skilled occupations. In towns where measurements had been recorded at multiple sites the mean value, after excluding instruments sited to investigate specific sources of pollution, was taken as the index of pollution for the town. The expected deaths for the standardised mortality ratios in 1950–53 were calculated by multiplying the England and Wales death rates at 12 sex-age groups in those years by the corresponding local populations in the 165 areas.

*Atmospheric deposit (undissolved).*—Table XI shows the correlations with the average amount of such deposit falling on unit area of the deposit gauge per month in 53 county boroughs. Coefficients exceeding 0·266 are significant at 1 in 20 probability level, those exceeding 0·345 at 1 in 100 level and those exceeding 0·437 at 1 in 1000 level. The coefficients with lung cancer, bronchitis and pneumonia after eliminating the effect of social factors are all highly significant. Cancer of the stomach is also related significantly with amount of deposit, but cancers of the oesophagus, intestine, breast and other sites show no appreciable correlation.

Table XII gives the coefficients in 74 districts of Lancashire and the West Riding of Yorkshire, and in the 71 of these which were urban in character. In the latter group coefficients exceeding 0·230 are significant at 1 in 20 level, those exceedings 0·300 at 1 in 100 level and those exceeding 0·376 at 1 in 1000 level. The partial coefficients when social index is held constant are highly significant for lung cancer and bronchitis, but with pneumonia they are small. Cancers of organs other than the lung show no appreciable correlations when social index is constant, but stomach cancer in females gives a significant coefficient in the 74 districts when persons per acre are made constant.

TABLE XI.—*Correlations in* 53 *County Boroughs between Amounts of Atmospheric Deposit* (*Undissolved*) *and Standardised Mortality from Cancer, Bronchitis and Pneumonia in* 1950–53, *Persons per Acre and Proportion of Males Aged* 14 *and Over in Social Classes IV–V in* 1931.

| | | 1st order coefficients | | | 2nd order with deposit (constant idices) | |
|---|---|---|---|---|---|---|
| | Sex | Deposit | Persons per acre | Social index | P.P.A. | Social |
| *Cancer* of | | | | | | |
| Lung (1950–54) | M. & F. | 0·536 | 0·650 | 0·391 | 0·531 | 0·477 |
| Oesophagus | M. | 0·114 | 0·192 | 0·228 | 0·009 | 0·061 |
| | F. | −0·193 | −0·103 | −0·133 | −0·176 | −0·171 |
| Stomach | M. | 0·414 | 0·077 | 0·360 | 0·408 | 0·344 |
| | F. | 0·383 | 0·111 | 0·401 | 0·374 | 0·301 |
| Intestine | M. & F. | 0·179 | 0·219 | 0·143 | 0·175 | 0·144 |
| Breast | F. | −0·171 | −0·075 | −0·337 | −0·158 | −0·078 |
| Other sites | M. | 0·224 | 0·419 | 0·028 | 0·094 | 0·226 |
| | F. | 0·097 | 0·126 | −0·025 | 0·072 | 0·109 |
| *Bronchitis* | M. | 0·621 | 0·346 | 0·504 | 0·579 | 0·570 |
| | F. | 0·540 | 0·245 | 0·379 | 0·511 | 0·483 |
| *Pneumonia* | M. | 0·503 | — | 0·464 | — | 0·431 |
| | F. | 0·685 | — | 0·487 | — | 0·647 |
| Persons per cre | — | 0·219 | — | — | — | — |
| Social index | — | 0·300 | — | — | — | — |

TABLE XII.—*Correlations in* 74 *Administrative Areas of Lancashire and the West Riding of Yorkshire between Amounts of Atmospheric Deposit* (*Undissolved*), *Standardised Mortality from Cancer, Bronchitis and Pneumonia in* 1950–53, *Persons per Acre and Proportion of Males Aged* 15 *and Over in Social Classes IV–V in* 1951.

| | | All areas with deposit data | | | Excluding Rural Districts | | |
|---|---|---|---|---|---|---|---|
| | | 1st order, with | | 2nd order. | 1st order | | 2nd order. |
| | Sex | Deposit | Persons per acre | Deposit (P.P.A. constant) | Deposit | Social index IV–V | Deposit (index constant) |
| *Cancer* of | | | | | | | |
| Lung (1950–54) | M. & F. | 0·353 | 0·611 | 0·260 | 0·358 | 0·125 | 0·400 |
| Stomach | M. | 0·221 | 0·140 | 0·189 | 0·206 | 0·365 | 0·030 |
| | F. | 0·226 | 0·014 | 0·232 | 0·329 | 0·399 | 0·158 |
| Breast | F. | 0·041 | −0·017 | 0·046 | 0·038 | −0·261 | — |
| Other sites | M. | 0·258 | 0·313 | 0·187 | 0·237 | 0·310 | 0·102 |
| | F. | 0·114 | 0·099 | 0·099 | 0·068 | 0·254 | −0·070 |
| *Bronchitis* | M. | 0·482 | 0·458 | 0·407 | 0·452 | 0·549 | 0·340 |
| | F. | 0·541 | 0·334 | 0·490 | 0·588 | 0·420 | 0·482 |
| *Pneumonia* | M. | 0·282 | — | — | 0·303 | 0·346 | 0·156 |
| | F. | 0·305 | — | — | 0·370 | 0·399 | 0·216 |
| Persons per acre | — | 0·305 | — | — | — | — | — |
| Social index IV–V | — | — | — | — | 0·498 | — | — |

Atmospheric particles large enough to fall into deposit gauges would seem unlikely to reach the lung, but abundance of them is usually accompanied by an excess of the finer particles which pass into smoke filters, and this may account partly for the high correlations with lung conditions. With stomach cancer there could be a direct agency, since such particles fall upon unprotected food and soil the hands, being then ingested.

TABLE XIII.—*Correlations in* 30 *County Boroughs between Amount of Smoke in Air, Standardised Mortality from Cancer, Bronchitis and Pneumonia in* 1950–53, *Persons per Acre and Proportion of Males Aged* 14 *and Over in Social Classes IV–V in the Population in* 1951.

| | | 1st order coefficients | | | 2nd order with smoke (constant indices) | |
|---|---|---|---|---|---|---|
| | Sex | Smoke | Persons per acre | Social index IV–V | P.P.A. | Class IV–V |
| *Cancer* of | | | | | | |
| Lung (1950–54) | M. | 0·494 | — | 0·448 | — | 0·326 |
| | F. | 0·491 | — | 0·468 | — | 0·309 |
| | M. & F. | 0·520 | 0·520 | 0·480 | 0·451 | 0·339 |
| Oesophagus | M. | 0·361 | 0·220 | 0·368 | 0·319 | 0·202 |
| | F. | 0·016 | 0·014 | — | 0·013 | — |
| Stomach | M. & F. | 0·685 | — | 0·539 | — | 0·546 |
| Intestine | M. & F. | 0·366 | — | 0·041 | — | 0·466 |
| Other sites | M. | 0·164 | 0·315 | 0·227 | 0·078 | 0·044 |
| | F. | 0·021 | — | −0·160 | — | 0·042 |
| *Bronchitis* | M. | 0·604 | 0·313 | 0·587 | 0·564 | 0·405 |
| | F. | 0·576 | 0·380 | 0·499 | 0·525 | 0·410 |
| *Pneumonia* | M. | 0·493 | 0·409 | 0·563 | 0·426 | 0·240 |
| | F. | 0·332 | 0·153 | 0·539 | 0·303 | 0·036 |
| Persons per acre | — | 0·297 | — | — | — | — |
| Social index IV–V | — | 0·569 | — | — | — | — |

*Smoke*.—Table XIII shows the correlations with average annual amounts of smoke per unit volume of air in 30 county boroughs where filters had been operating. Coefficients exceeding 0·349 are significant at 1 in 20 level of probability, those exceeding 0·449 at 1 in 100 level and those exceeding 0·554 at 1 in 1000 level. The coefficient with lung cancer when population density is constant is 0·451 and when social index is constant it is 0·339. Bronchitis and cancers of the stomach and intestine also give significant correlations after eliminating the social factors, but the pneumonia figures are not so definite. Cancer of the oesophagus gives coefficients of doubtful significance and cancer of other sites shows no associations with smoke.

Table XIV gives coefficients in 45 districts of Lancashire and the West Riding where filters had been operating, and in 42 after excluding rural districts, those exceeding 0·287 being significant at 1 in 20 probability level and those exceeding 0·372 at 1 in 100 level. The correlations with lung cancer remain highly significant when social index is constant whichever criterion is used. Bronchitis and pneumonia coefficients are just below significance level. Cancers of the stomach, and of the breast and other sites in females show no appreciable associations with smoke, but other cancer in males gives a significant coefficient in the urban areas.

TABLE XIV.—*Correlations in* 45 *Administrative Areas of Lancashire and the West Riding of Yorkshire between Amounts of Smoke in Air, Standardised Mortality from Cancer, Bronchitis and Pneumonia in* 1950–53, *and Proportions of Males in Social Classes IV–V in* 1951.

| | | All areas with smoke data | | | Excluding Rural Districts | | |
|---|---|---|---|---|---|---|---|
| | | 1st order | | 2nd order. | 1st order | | 2nd order. |
| | Sex | Smoke | Class V index | Smoke (index constant) | Smoke | Class IV and V index | Smoke (index constant) |
| *Cancer* of | | | | | | | |
| Lung (1950–54) | M. & F. | 0·558 | 0·577 | 0·353 | 0·531 | 0·149 | 0·519 |
| Stomach | M. | 0·260 | 0·056 | 0·288 | 0·230 | 0·385 | 0·091 |
| | F. | 0·230 | −0·017 | 0·310 | 0·225 | 0·483 | 0·041 |
| Breast | F. | −0·002 | −0·160 | 0·125 | −0·006 | −0·319 | 0·139 |
| Other sites | M. | 0·345 | 0·342 | 0·181 | 0·319 | −0·026 | 0·390 |
| | F. | 0·034 | −0·037 | 0·073 | −0·001 | 0·068 | −0·030 |
| *Bronchitis* | M. | 0·440 | 0·389 | 0·280 | 0·435 | 0·443 | 0·315 |
| | F. | 0·320 | 0·258 | 0·276 | 0·307 | 0·253 | 0·232 |
| *Pneumonia* | M. | 0·442 | 0·453 | 0·232 | 0·406 | 0·458 | 0·274 |
| | F. | 0·423 | 0·314 | 0·303 | 0·426 | 0·460 | 0·299 |
| Social class IV–V indices | — | 0·361 | — | — | 0·397 | — | — |
| V | — | 0·619 | — | — | 0·689 | — | — |

TABLE XV.—*Correlations in* 40 *Areas of Greater London between Amount of Smoke in Air, Standardised Mortality in* 1950–53 *from Cancer, Bronchitis, and Pneumonia, and Proportion of Males Aged* 15 *and Over in Social Classes IV and V in the Population.*

| | | 1st order coefficients | | | 2nd order with smoke (constant indices) | |
|---|---|---|---|---|---|---|
| | | | Social indices | | | |
| | Sex | Smoke | IV–V | V only | IV–V | V only |
| *Cancer* of | | | | | | |
| Lung | M. | 0·408 | 0·684 | 0·695 | 0·221 | 0·228 |
| | F. | 0·386 | 0·368 | 0·371 | 0·284 | 0·292 |
| Stomach | M. | 0·330 | 0·809 | — | 0·032 | — |
| | F. | 0·203 | 0·579 | — | −0·025 | — |
| All other sites | M. | 0·164 | 0·514 | — | −0·042 | — |
| | F. | 0·325 | 0·278 | — | 0·246 | — |
| *Bronchitis* | M. | 0·454 | 0·773 | — | 0·269 | — |
| | F. | 0·566 | 0·698 | — | 0·299 | — |
| *Pneumonia* | M. | 0·530 | 0·695 | — | 0·410 | — |
| | F. | 0·466 | 0·584 | — | 0·322 | — |
| Social class IV–V indices | — | 0·384 | — | — | — | — |
| V | — | 0·368 | — | — | — | — |

Table XV shows the correlations in 40 areas of Greater London where smoke data were available, the number comprising 15 districts in the Outer Ring, 24 Metropolitan Boroughs and a central area covering Holburn, City, Finsbury and Shoreditch. In view of the great daily movement of people, particularly of men, from one district to another it would hardly be surprising to find little or no correlation between mortality of residents and the average smoke density in the area of residence when social differences have been allowed for. Coefficients exceeding 0·304 are significant at 1 in 20 level, and the lung cancer and bronchitis correlations are slightly below this level, though the pneumonia figures are significant for males and females. Stomach cancer shows no associations with smoke nor does cancer of other sites in males, but for other sites in females there is a small but not significant correlation. The Greater London results are in general accord with those from the other groups of areas, though as was expected the associations with smoke are weaker.

### SUMMARY

Lung cancer mortality is strongly correlated with smoke density in the atmosphere in 26 areas of Northern England and Wales, in 45 districts of Lancashire and the West Riding of Yorkshire, and in 30 county boroughs, whilst similar though weaker correlations are found within Greater London. These relations are only partially explicable by social differences in the populations concerned. Bronchitis and pneumonia in males and bronchitis also in females show similar strong correlations with smoke. Cancers of the stomach and intestine are related significantly with smoke in the county boroughs, and cancers of sites other than lung and stomach in males are so related in the other groups of areas. In females cancers of the breast and other organs show no association with smoke. Pollution by larger particles is related significantly with lung and stomach cancers, bronchitis and pneumonia in 53 county boroughs and with lung cancer and bronchitis in 74 districts of Lancashire and the West Riding.

In the 26 localities the smoke samples were analysed in respect of polycyclic hydrocarbons and a statistical process of successive elimination was applied to discover which hydrocarbon was responsible for the smoke correlation with mortality rates. For lung cancer and bronchitis 3 : 4 benzopyrene emerges clearly as the substance of prime importance, with 1 : 12 benzoperylene contributing weakly for lung cancer, but for pneumonia 3 : 4 benzopyrene is apparently not important. The composite group of other cancers in males is correlated with several hydrocarbons, but cancers of the breast and other sites in females show no relations with any of them.

In 23 localities spectrographic analyses for 13 trace elements were made and a similar process of successive elimination was applied to those which showed appreciable correlations with mortality rates. For lung cancer beryllium and molybdenum emerge as the elements of most consequence, with arsenic, zinc and vanadium showing weaker associations. For bronchitis molybdenum appears to be the important element in both sexes whilst in males beryllium, arsenic, vanadium and zinc may also be concerned as for lung cancer. For pneumonia beryllium emerges as the important element in both sexes, with vanadium also concerned in males. With other cancer in males beryllium, molybdenum and vanadium show associations, but breast and other cancers in females show no relations with any element.

My thanks have been expressed in previous reports to the many people who contributed to the early stages of this co-operative study. I am indebted particularly to Mr. B. T. Commins of the Medical Research Council Group at the Dunn Laboratory for the analytical work on the hydrocarbons and to Mr. K. V. Aubrey at the Fuel Research Station of the Department of Scientific and Industrial Research for the spectrographic work, and have to thank Mr. R. E. Waller and

Dr. E. T. Wilkins of those departments for help and advice, the General Register Office and County Medical Officers for supplying mortality data and the Health Officers of all the districts where instruments were installed for their co-operation.

This work was supported by grants from the British Empire Cancer Campaign and Medical Research Council.

### REFERENCES

CLIFTON, M., KERRIDGE, D., MOULDS, W., PEMBERTON, J. AND DONOGHUE, J. K.—(1959) *Int. J. Air Pollution*, **2**, 188.

COMMINS, B. T.—(1958) *Analyst*, **83**, 386.

HUEPER, W. C.—(1957) ' Cancer ', Vol. 1. London (Butterworth).

REGISTRAR GENERAL.—(1936) Statistical Review for 1934, Text.

SJOBERG, S. G.—(1950) *Acta med. scand.* (Suppl. 238), **138**, 1.—(1956) *Ibid.*, **154**, 381.

STOCKS, P.—(1955) *J. Fac. Radiol., Lond.*, **6**, 166.—(1958*a*) Supplement to 35th *Rep. Brit. Emp. Cancer Campgn.*—(1958*b*) *Int. J. Air Pollution*, **1**, 1.—(1959) *Brit. med. J.*, i, 74.

*Idem* and CAMPBELL, J. M.—(1955) *Ibid.*, ii, 923.

*Idem*, COMMINS, B. T. AND AUBREY, K. V.—(1960) *Int. J. Air Pollution*, **3** (in press).

*Idem* AND DAVIES, R. I.—(1960) *Brit. J. Cancer*, **14**, 8.

TOBACCO MANUFACTURERS' STANDING COMMITTEE.—(1959) ' Statistics of Smoking ' (2nd edition). London.

WYERS, H.—(1946) *Brit. J. industr. Med.*, **3**, 177.


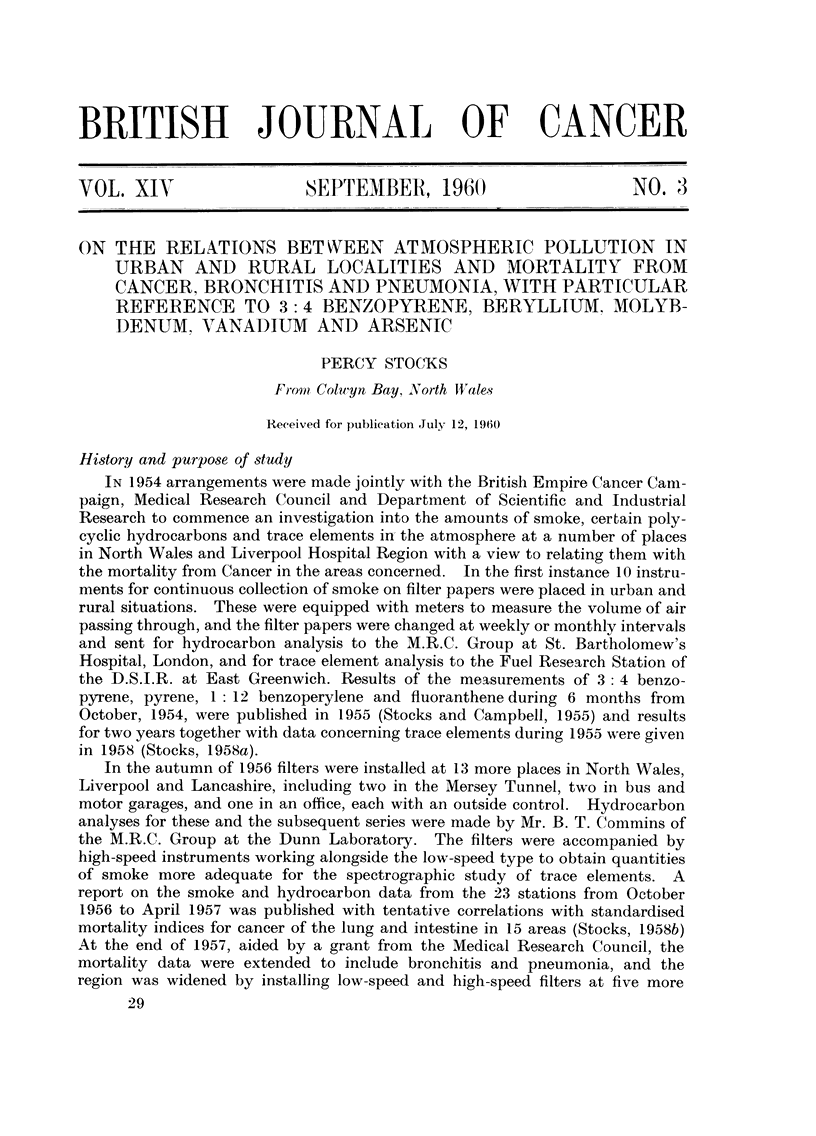

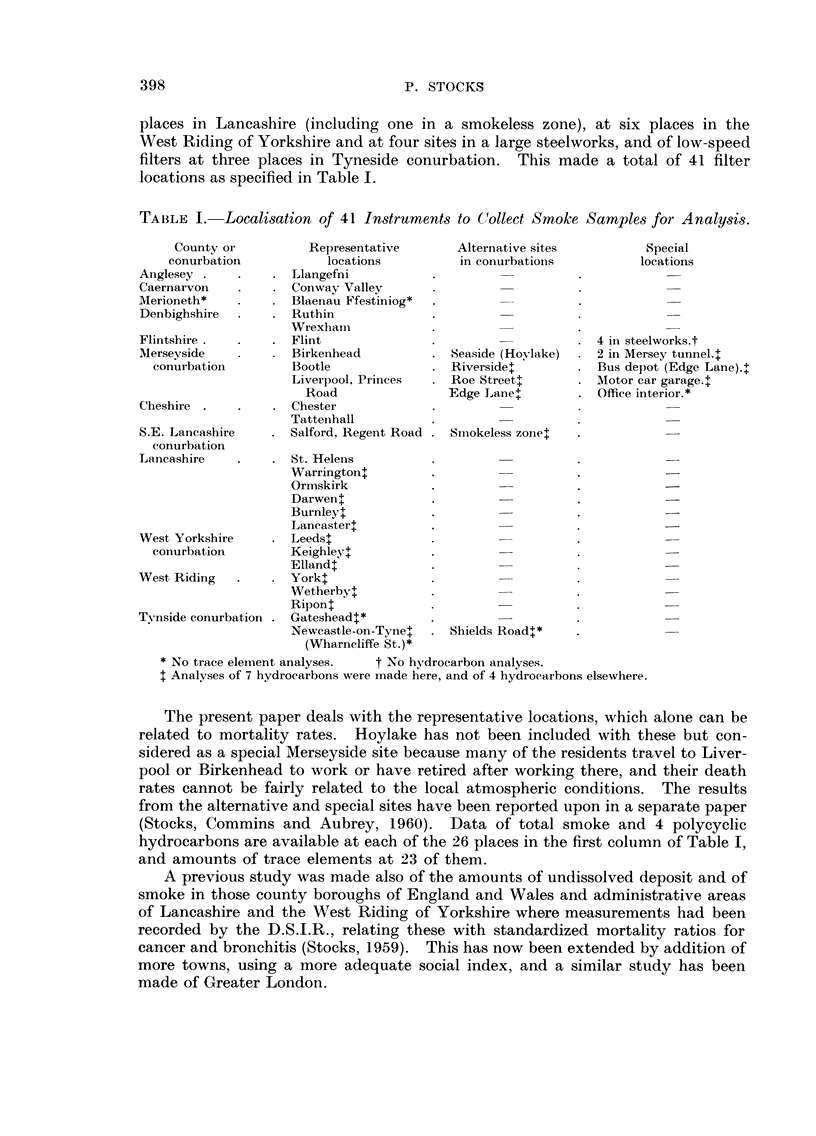

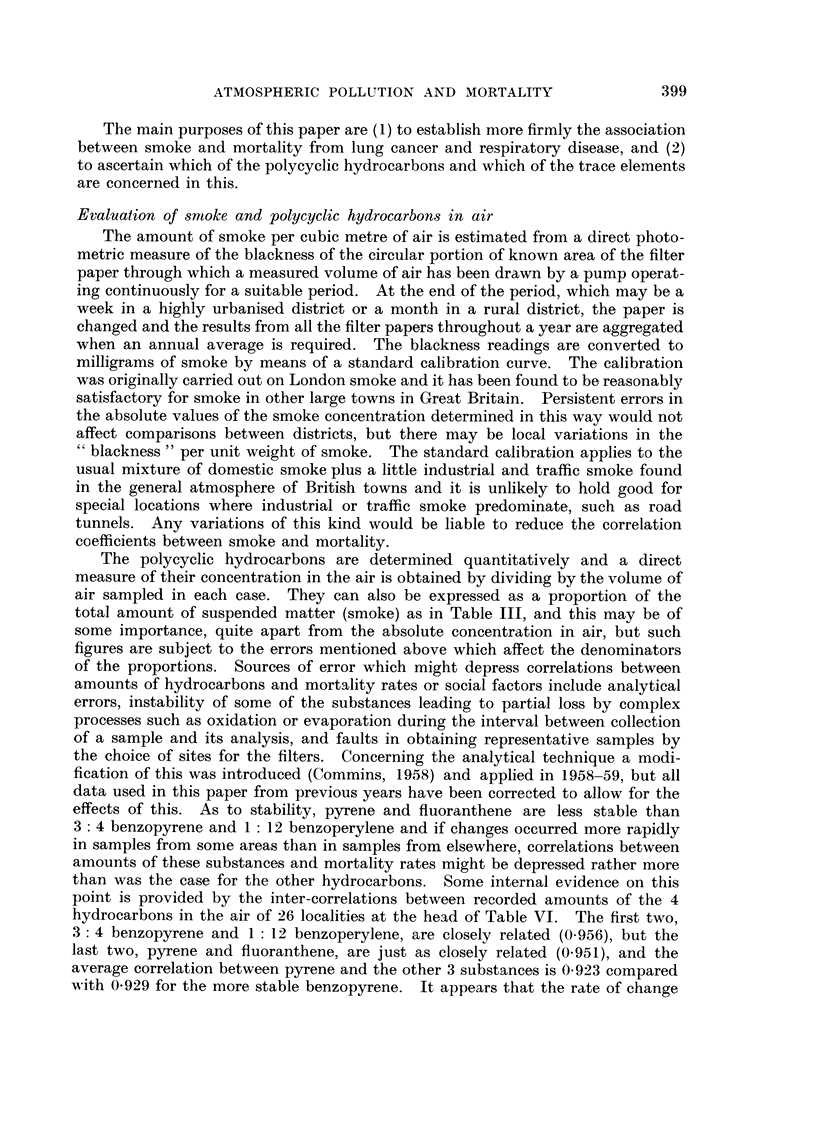

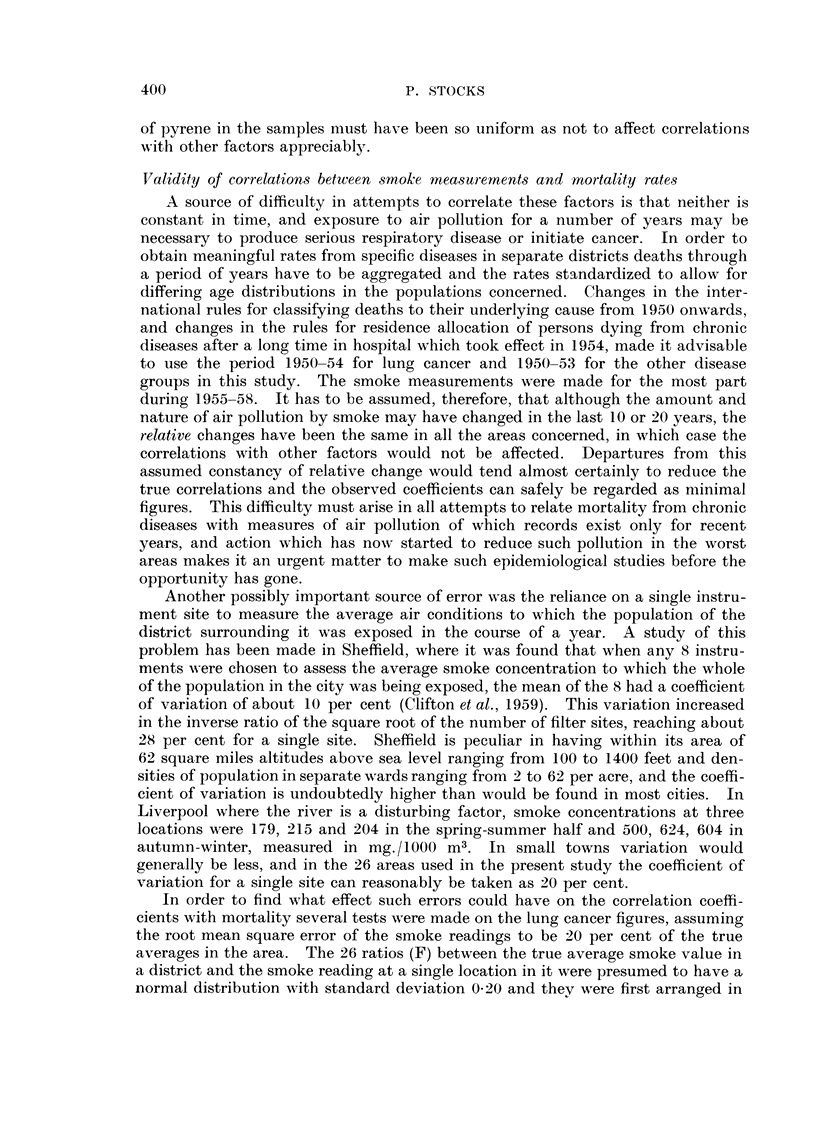

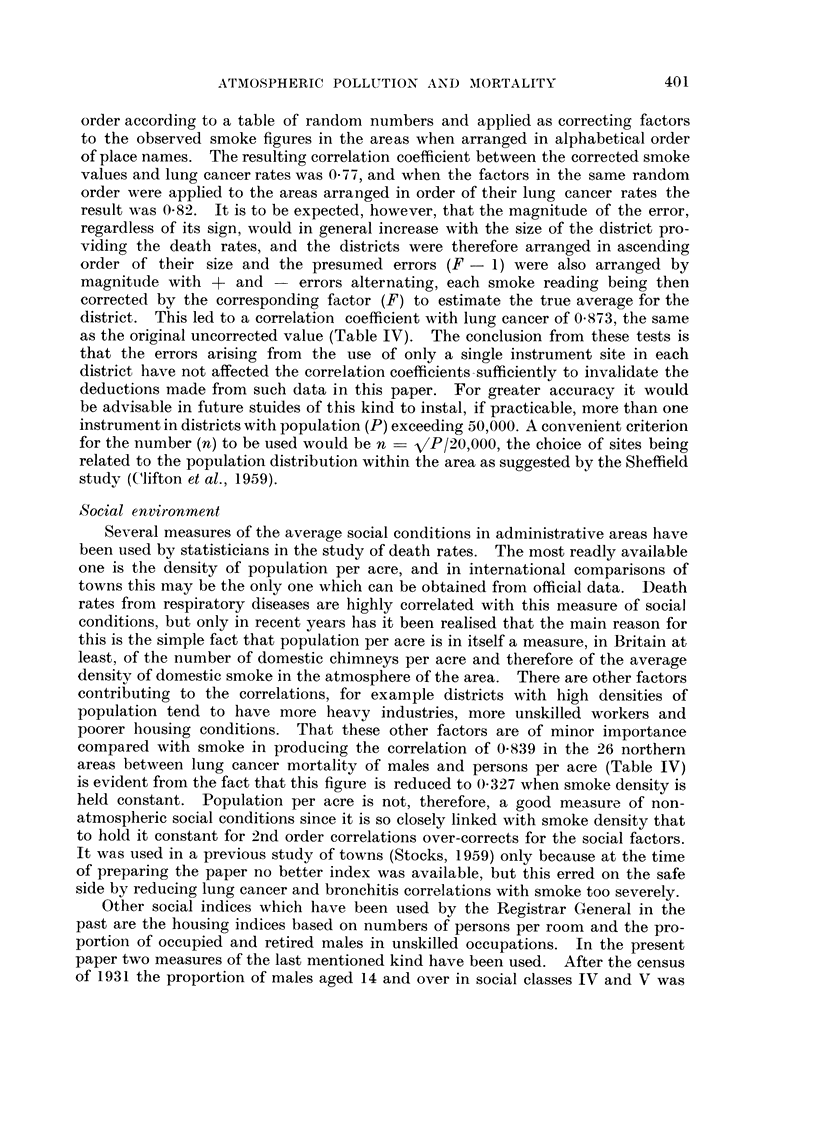

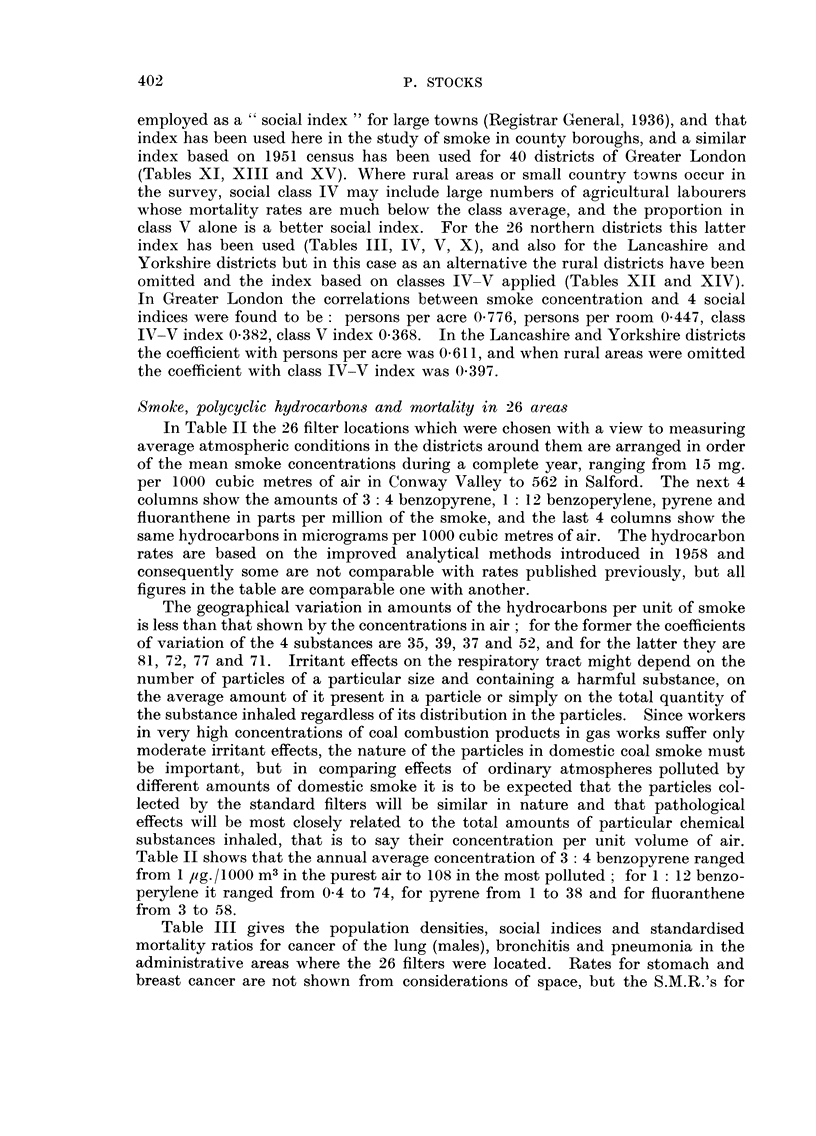

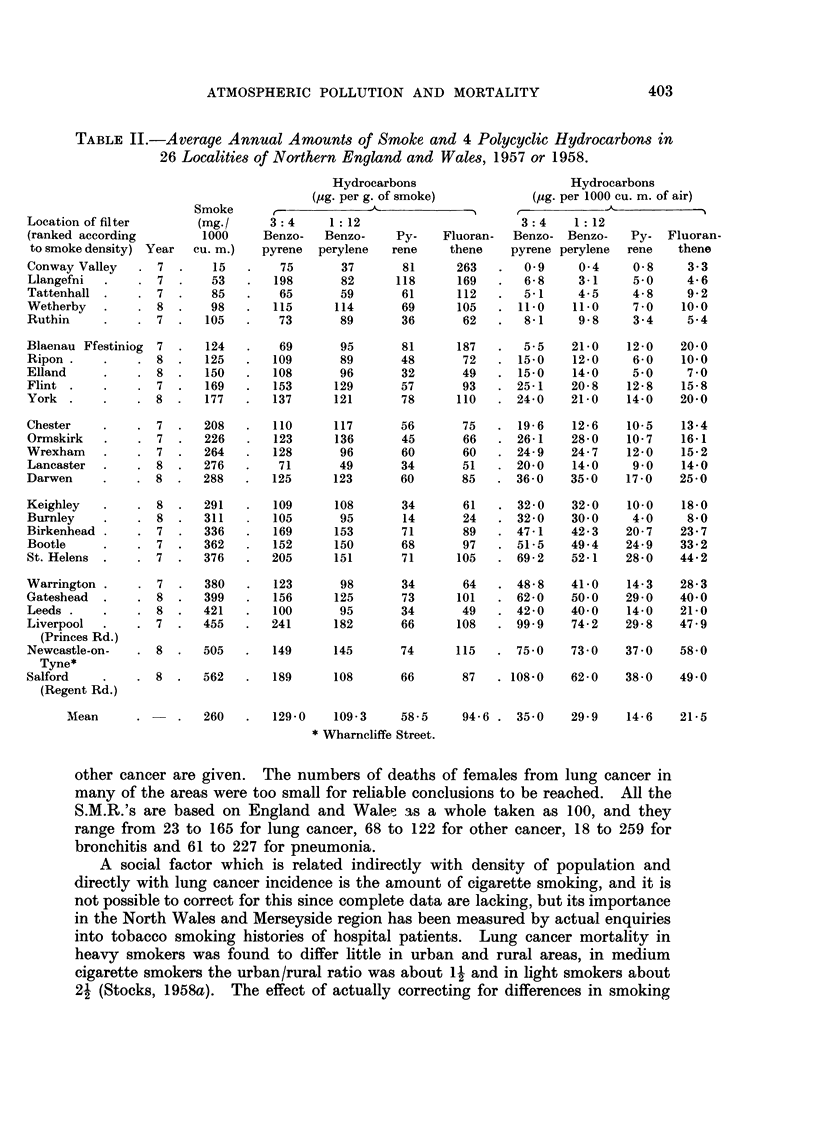

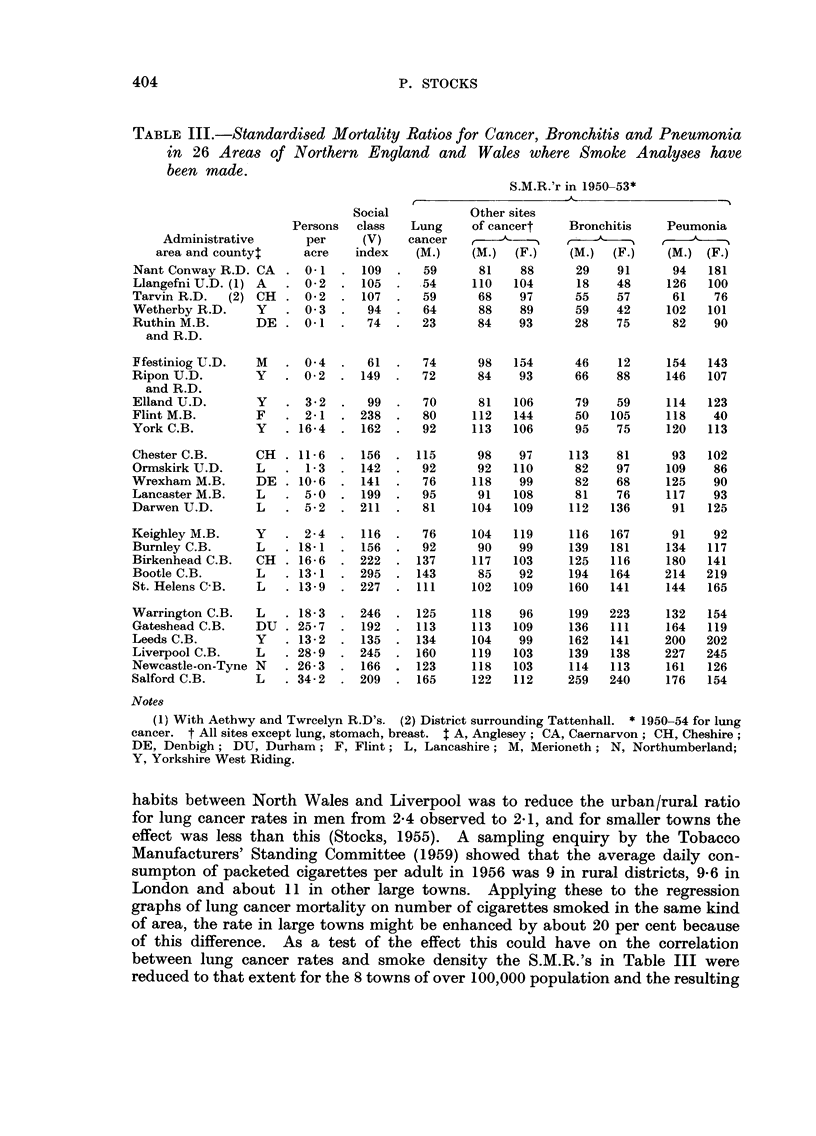

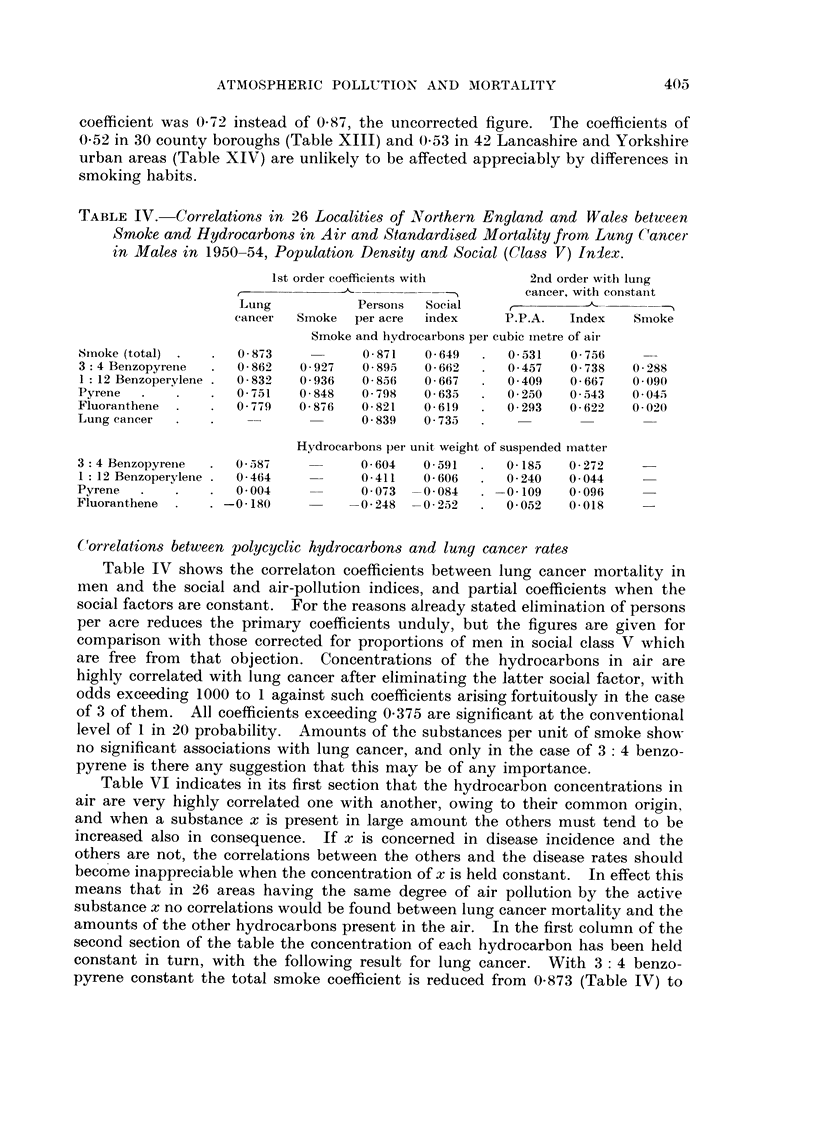

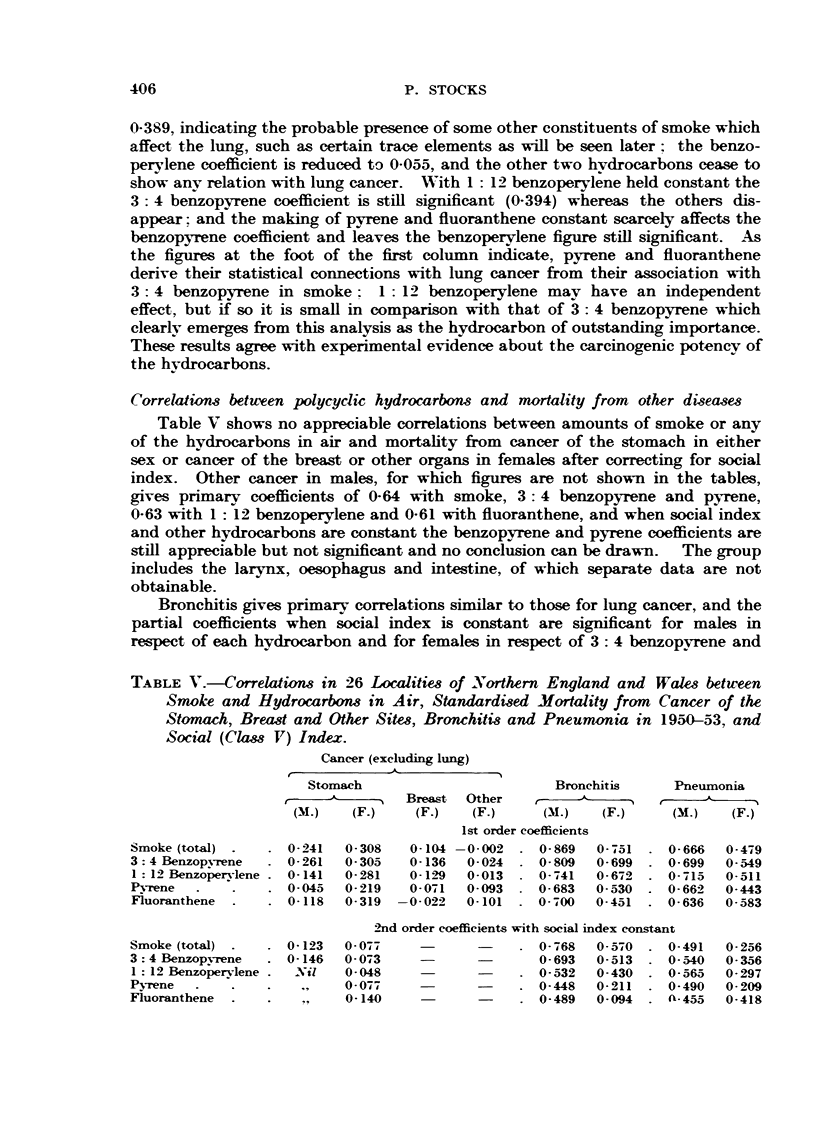

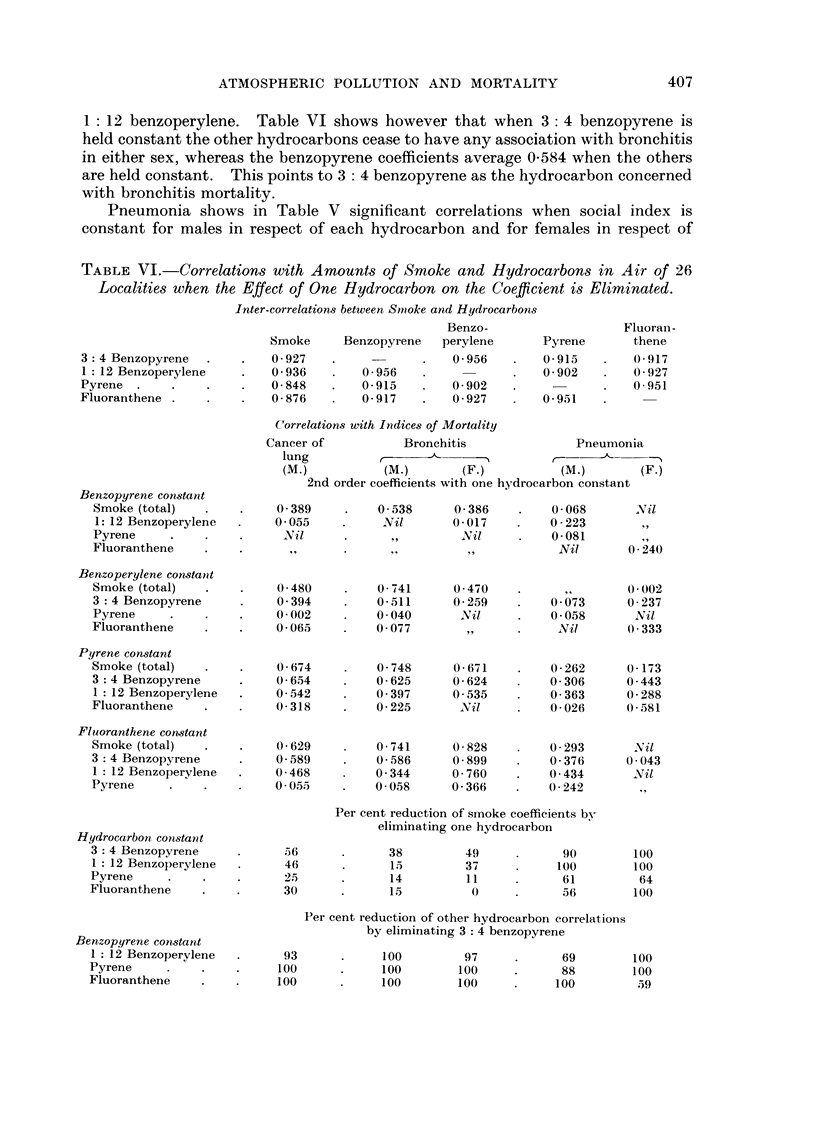

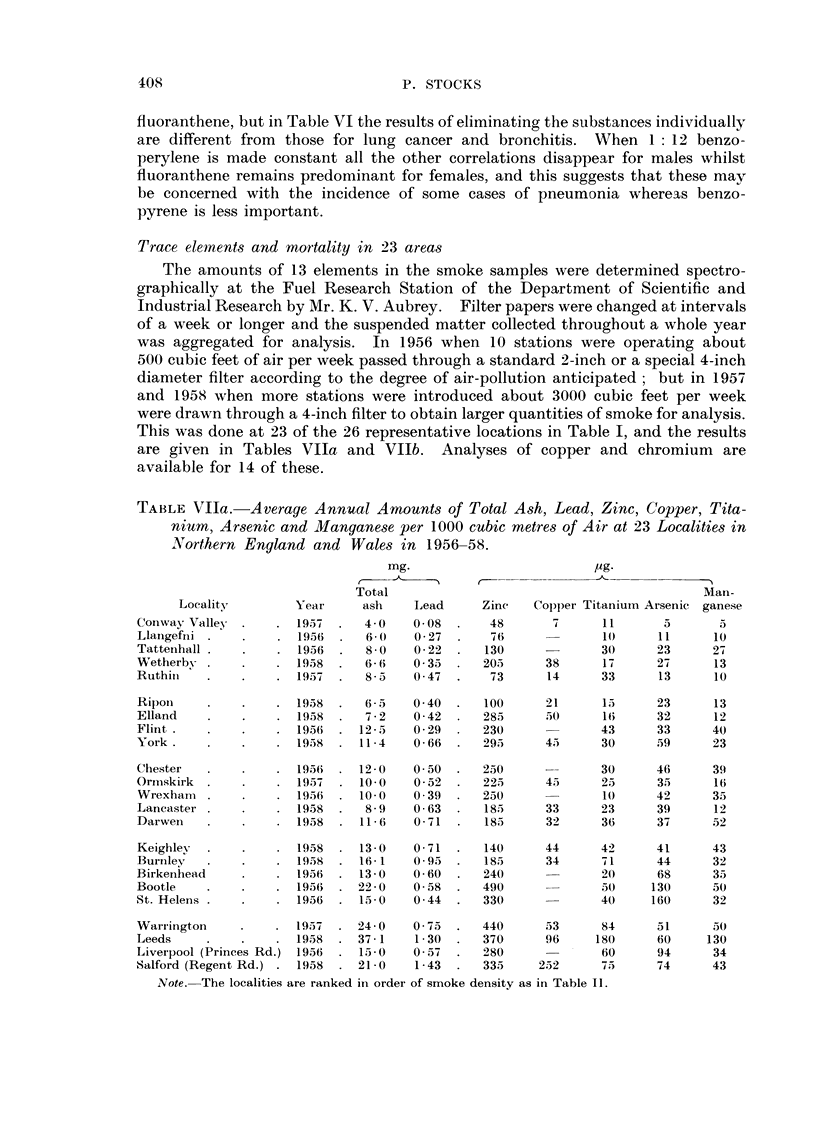

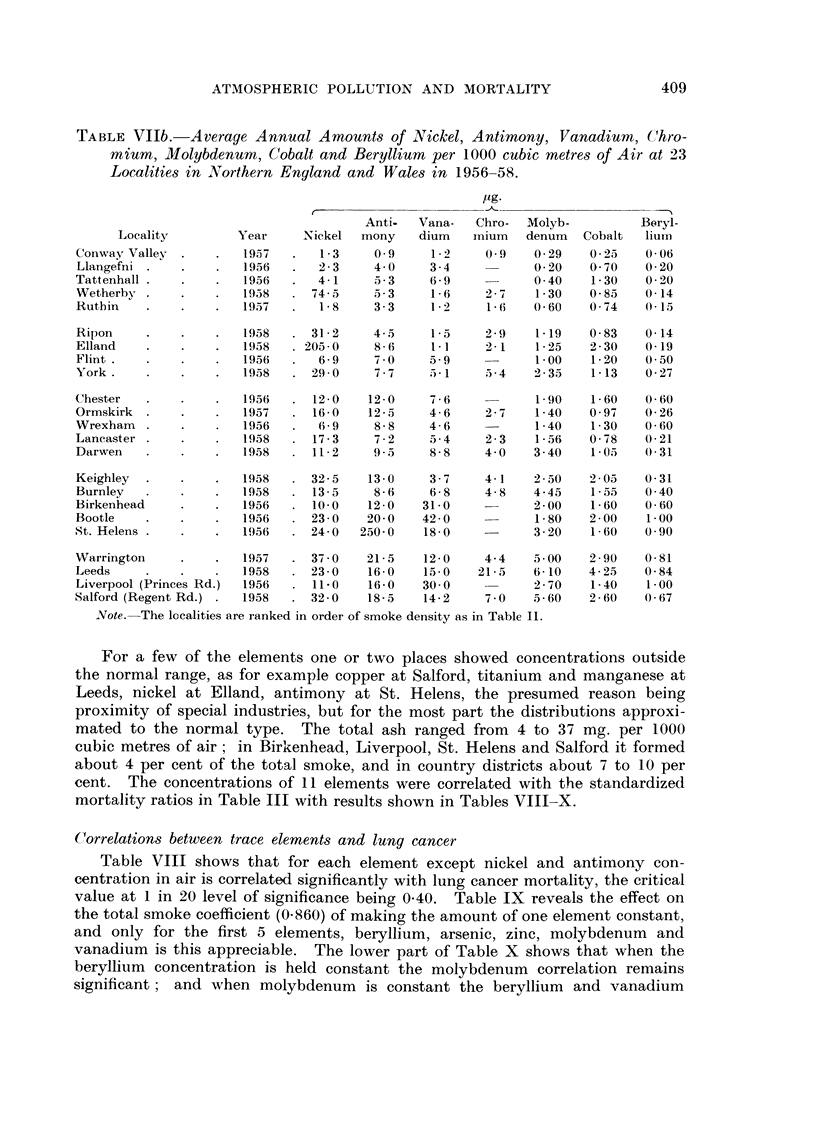

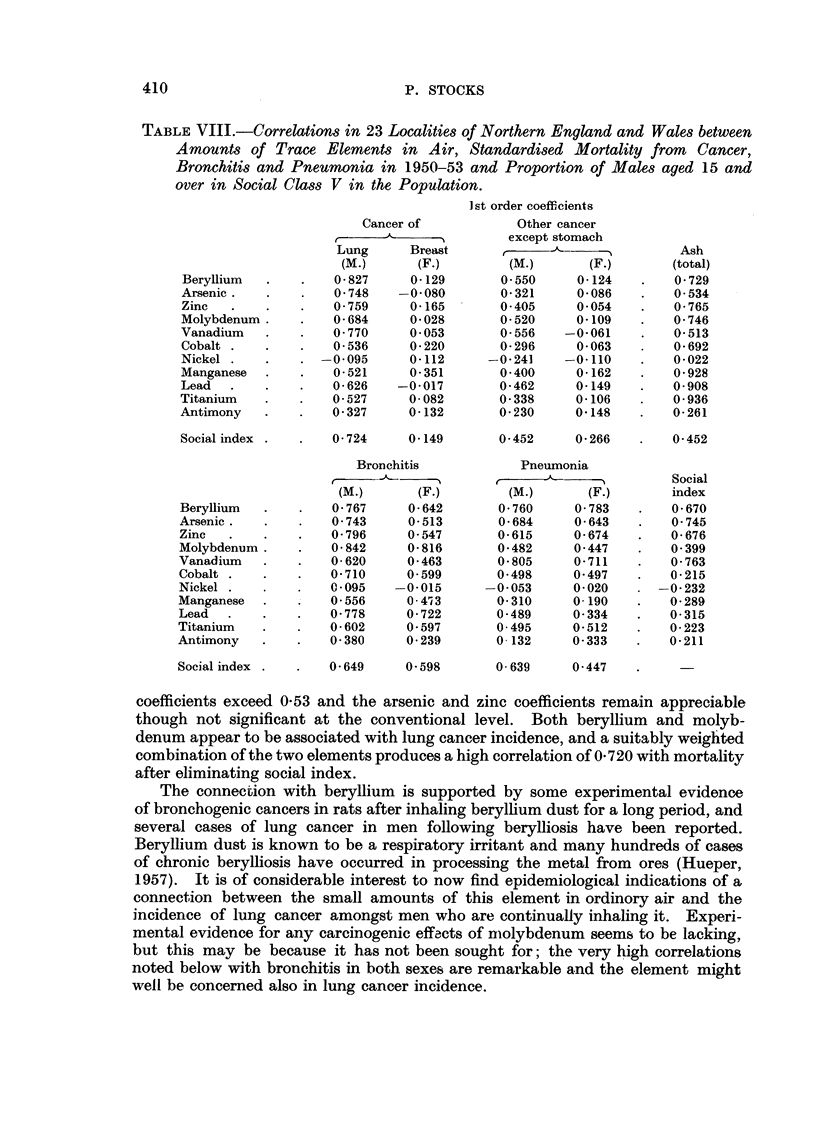

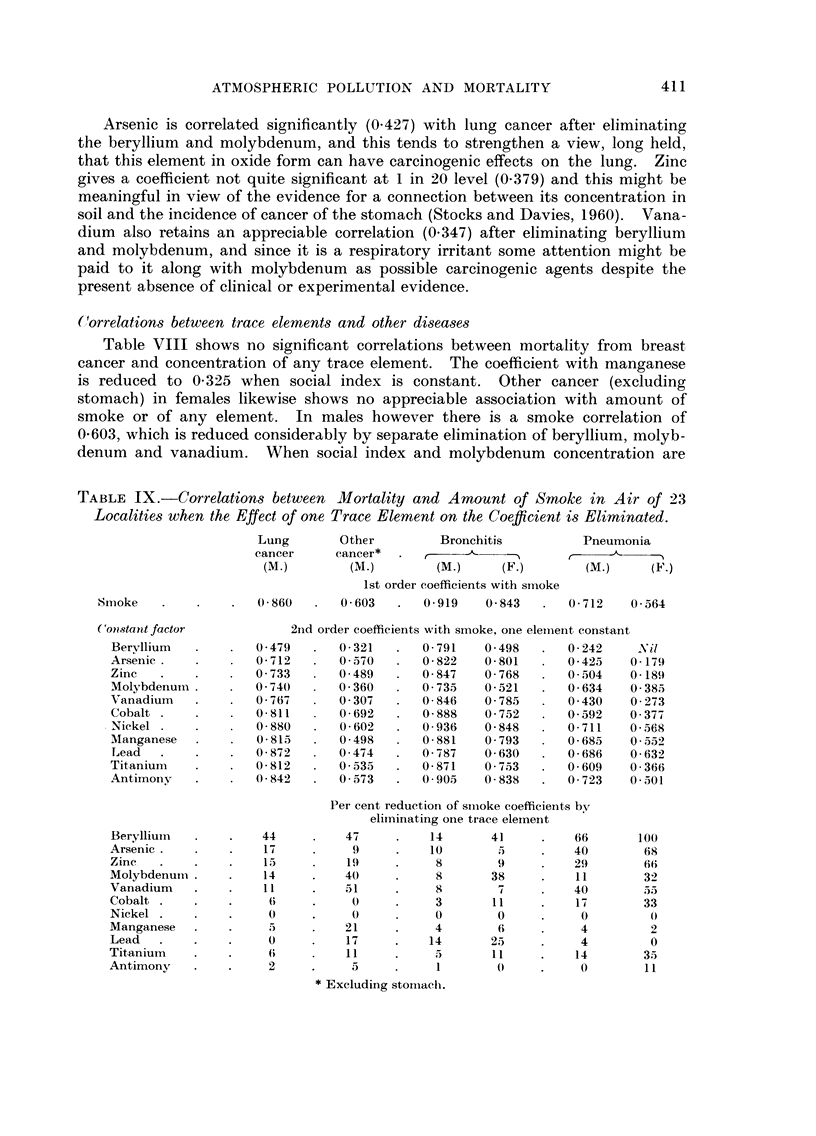

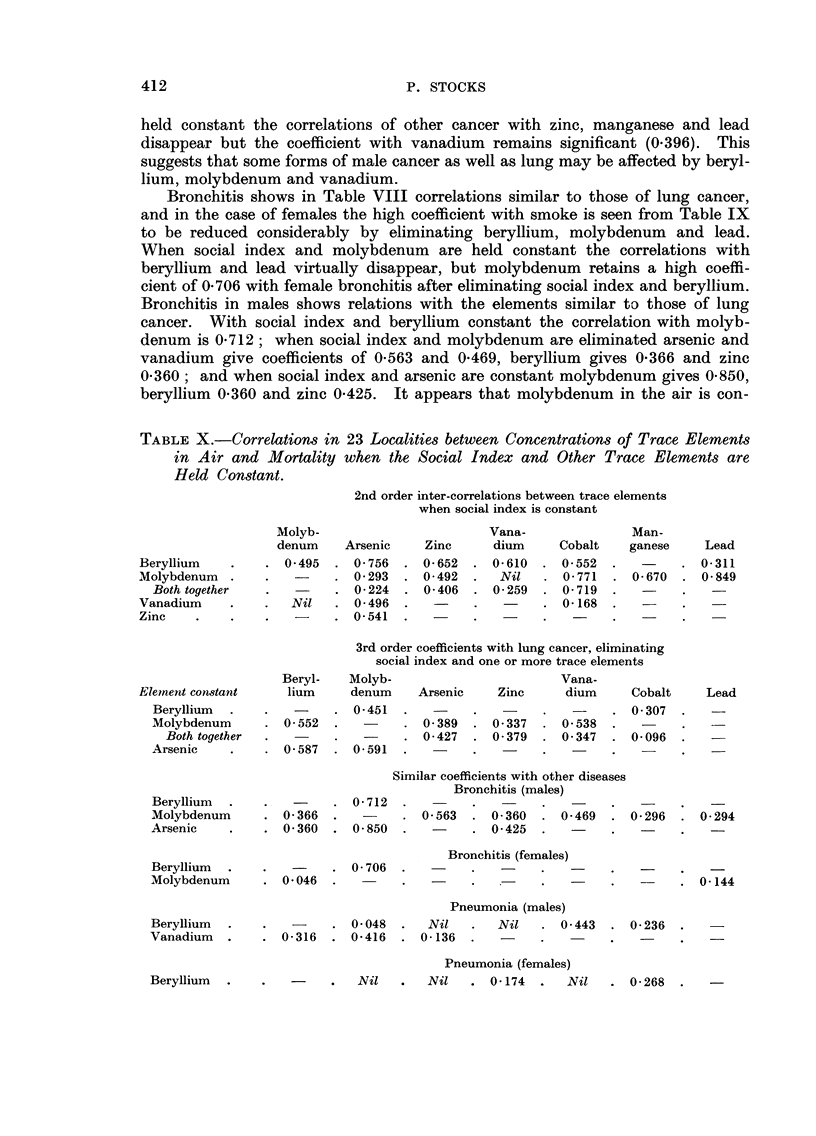

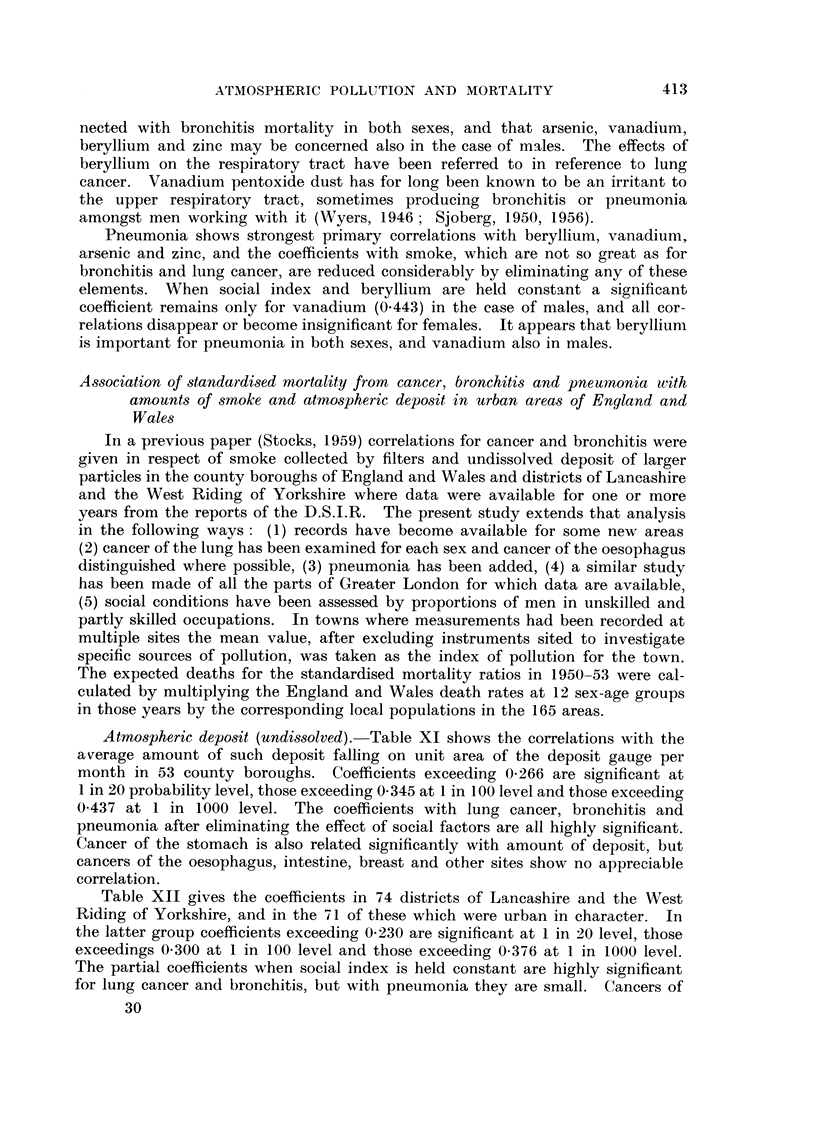

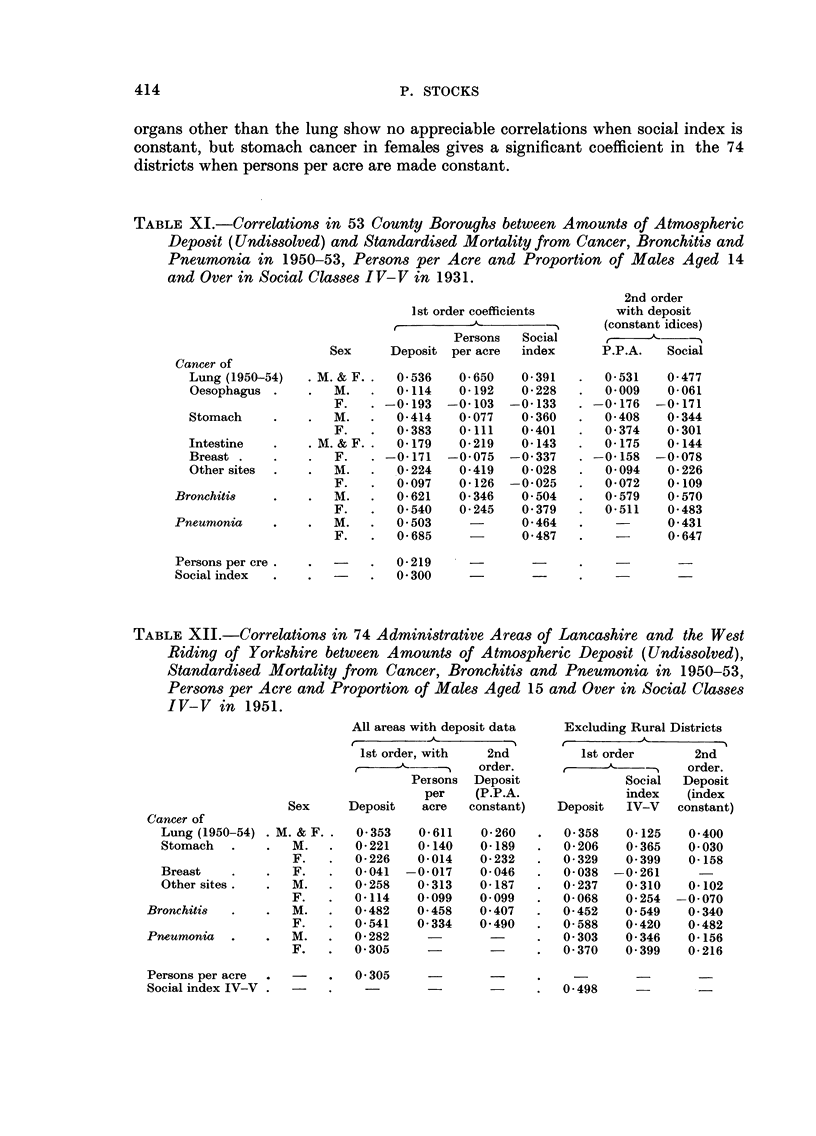

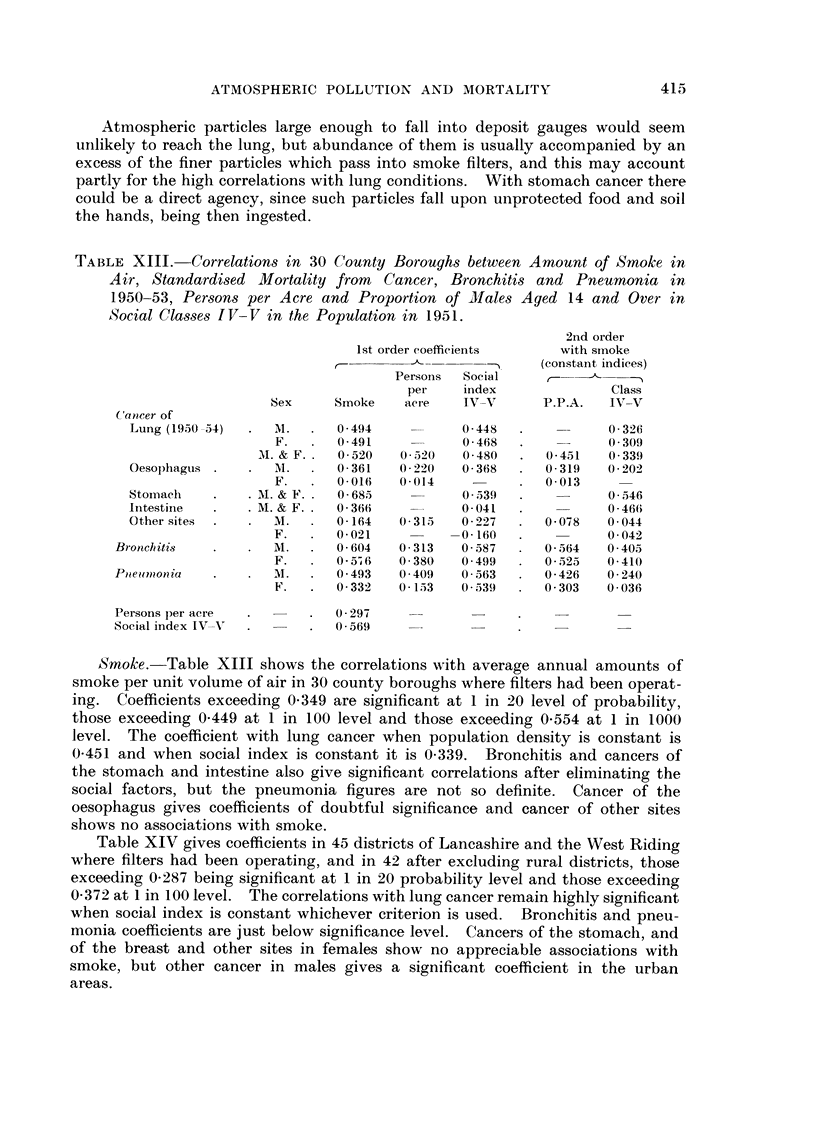

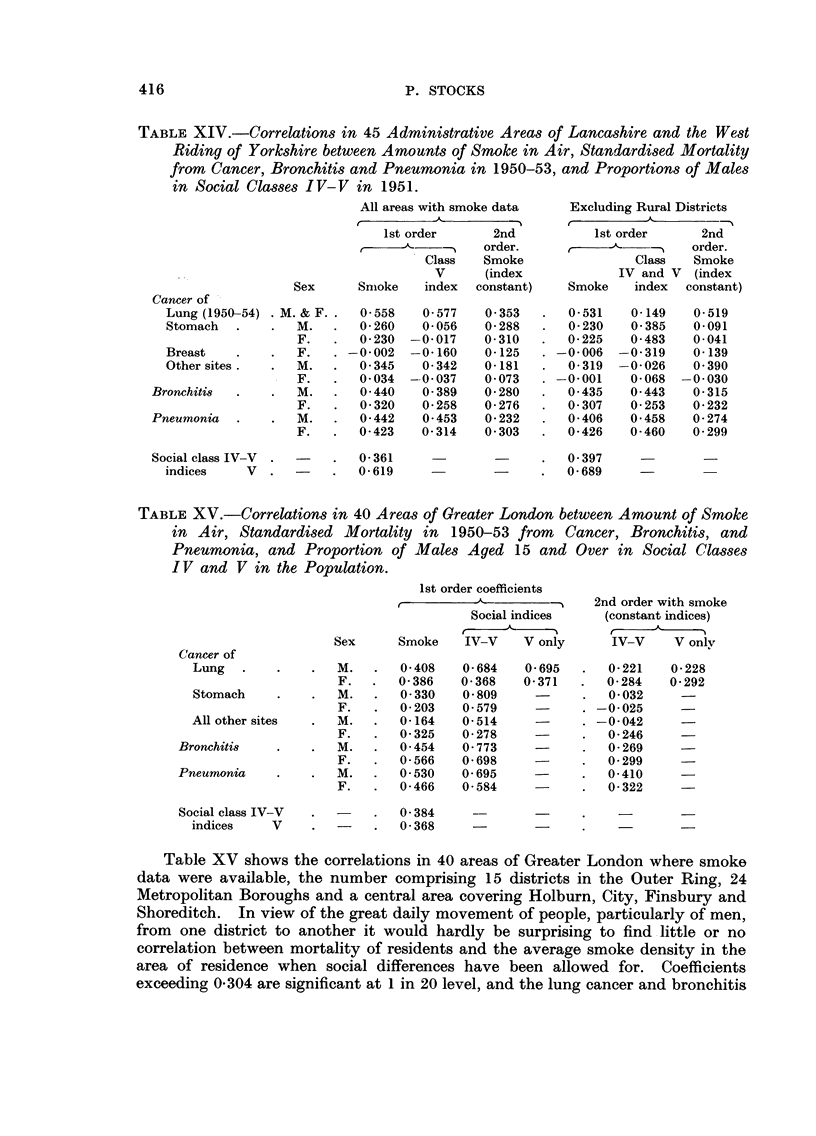

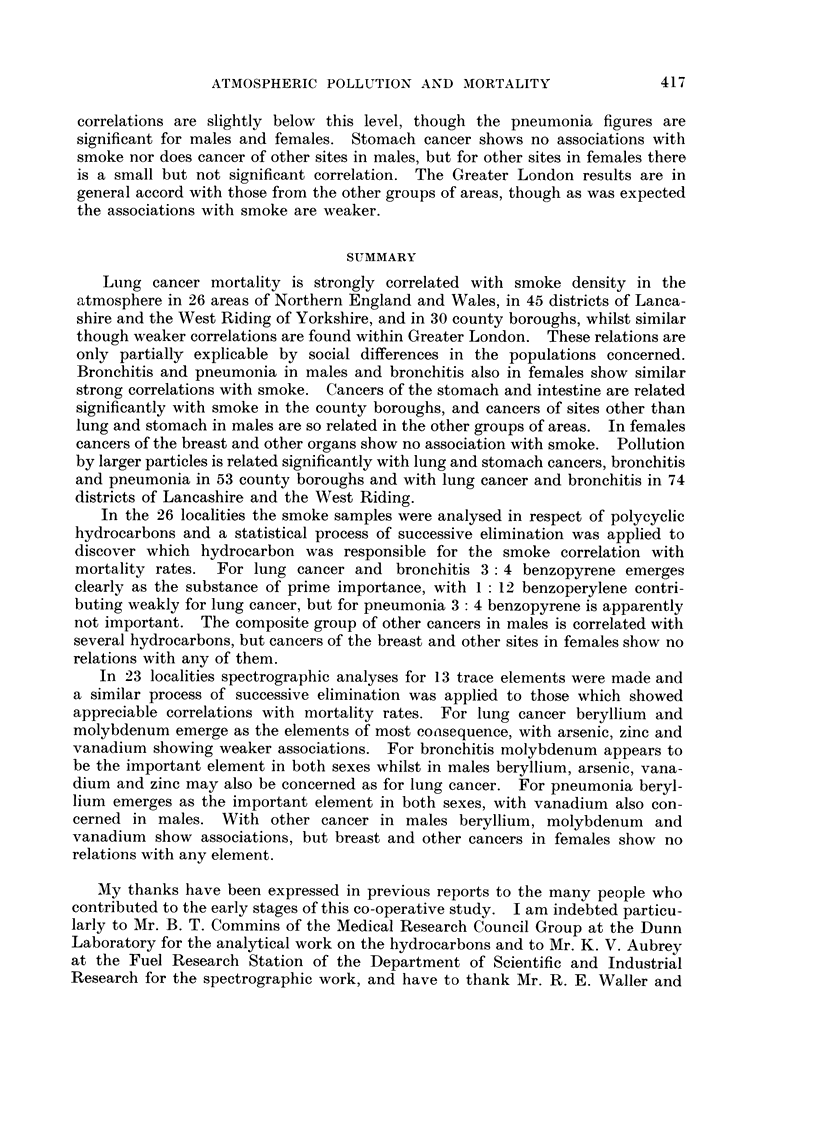

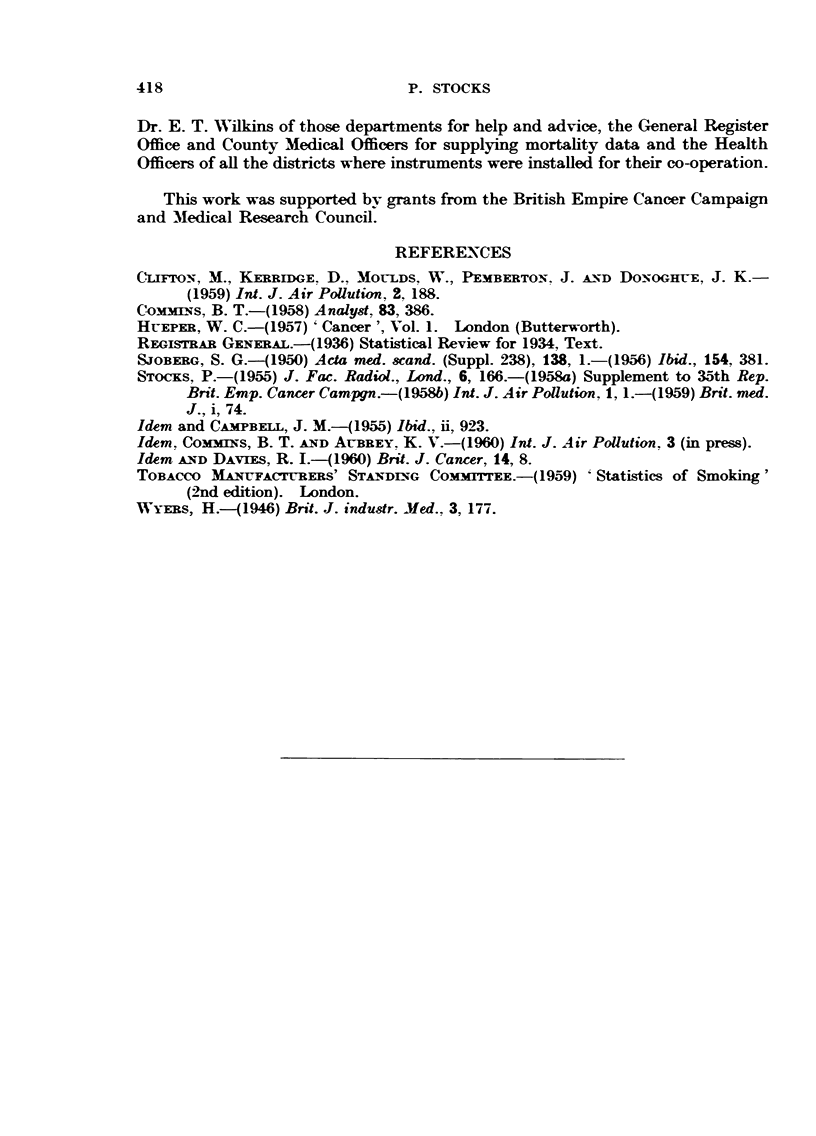


## References

[OCR_02590] CLIFTON M., KERRIDGE D., MOULDS W., PEMBERTON J., DONOGHUE J. K. (1959). The reliability of air pollution measurements in relation to the siting of instruments.. Int J Air Pollut.

